# Computing Mathematical Functions using DNA via Fractional Coding

**DOI:** 10.1038/s41598-018-26709-6

**Published:** 2018-05-29

**Authors:** Sayed Ahmad Salehi, Xingyi Liu, Marc D. Riedel, Keshab K. Parhi

**Affiliations:** Department of Electrical and Computer Engineering, University of Minnesota, 200 Union St. S.E., Minneapolis, MN 55455 USA

## Abstract

This paper discusses the implementation of mathematical functions such as exponentials, trigonometric functions, the sigmoid function and the perceptron function with molecular reactions in general, and DNA strand displacement reactions in particular. The molecular constructs for these functions are predicated on a novel representation for input and output values: a fractional *encoding*, in which values are represented by the relative concentrations of two molecular types, denoted as type-1 and type-0. This representation is inspired by a technique from digital electronic design, termed *stochastic* logic, in which values are represented by the probability of 1’s in a stream of randomly generated 0’s and 1’s. Research in the electronic realm has shown that a variety of complex functions can be computed with remarkably simple circuitry with this stochastic approach. This paper demonstrates how stochastic electronic designs can be translated to molecular circuits. It presents molecular implementations of mathematical functions that are considerably more complex than any shown to date. All designs are validated using mass-action simulations of the chemical kinetics of DNA strand displacement reactions.

## Introduction

Molecular computing holds the promise for transforming research in areas such as disease monitoring and drug delivery. Since early, pioneering work by Adleman^1^, the field has evolved significantly. A particularly promising strategy for molecular computation is based on the mechanism of DNA strand displacement^2–6^.

Various computational structures have been proposed for DNA-based systems, as well as in other contexts. Simple logic primitives, such as AND, OR, NAND, NOR, and XOR have been demonstrated^7–14^. These circuits have been used as building blocks for both digital signal processing^15–20^, and mixed-signal (i.e., analog and digital) computation^17,21^. Using these simple circuits, complex genetic circuits have been constructed to perform computation in cells^22^. To automate the design of genetic and DNA circuits, computer-aided design (CAD) systems have been presented^22,23^. There has been interest in activating and inhibiting pathways by “filtering” concentrations of molecular types in different frequency bands^24–26^.

The theory of computing with abstract chemical reactions, termed chemical reaction networks (CRNs), has evolved into a bona fide *computer programming framework*. Work on CRNs includes programs for computing different sorts of mathematical functions such as polynomials^27,28^, and logarithms^29,30^.

In prior work, different approaches to compute complex functions such as exponentials and sigmoids have been presented. For example, it has been shown that CRNs describing covalent modification cycle can realize exponential, logarithm and sigmoid functions^29,31^. The hyperbolic regime can be used to realize exponential and logarithm while ultrasensitive regime can be used to realize sigmoid function. The CRNs in^29,31^ describe analog behavior of the system while the CRNs described by the proposed approach describe digital behavior. In^29,31^, each region of operation is described by a specific input-output characteristic. This implies that the exponential and sigmoid functions are realized for specific ranges of input concentrations. Furthermore, the sigmoid function in^29^ describes a hard-limit response. On the other hand, the proposed approach realizes digital circuits and the function behavior is not limited to a specific range of input concentrations (only the ratio of two concentrations used to represent a variable is important not their concentrations).

This paper presents a method for designing CRNs that compute a wide range of mathematical functions, ranging from simple to complex. The building blocks in the proposed methodology are units composed of four chemical reactions. All chemical reactions in the proposed system have exactly two reactants. Such bimolecular chemical reactions can be implemented as DNA strand-displacement reactions in a robust way^32^. Thus, our method provides a systematic way to design DNA reactions that compute mathematical functions. These computational constructs are central to the topic of perceptrons, that represent simple machine learning algorithm.

Machine learning classifiers have become ubiquitous in the computational sciences. Their physical realization using different technologies has been considered^33,34^. Molecular implementations of machine learning classifiers could have important applications. One can imagine instances where *inference* and *learning* might be an integral part of tasks such as biochemical sensing. For example, genetic logic circuits for cell classification can sense features of mRNAs; they can detect their expression patterns and selectively respond to specific cell types^35–38^. Such circuits could enable the production of personalized “smart” drugs that target specific diseases for specific patients^39^.

Past work on neural computation with molecular reactions includes^40–44^. As early theoretical research on the topic, Hjelmfelt *et al*. presented chemical reactions that, based on the ordinary differential equations of mass action kinetics model, can emulate so-called McClulloch-Pitts neurons^44^. These chemical neurons can be coupled together to build chemical neural networks or finite-state machine^45^. Also in a theoretical vein, Mills *et al*. described a DNA implementation of a Hopfield neural network as well as a DNA implementation of a multi-layer perceptron^46,47^. The authors speculated that networks containing as many as 10^9^ neurons might be feasible.

Laplante *et al*. performed pattern recognition with chemical (as opposed to biomolecular) reactions, in a continuous flow stirred tank reactors^48^. Lim *et al*. implemented pattern recognition with differentially-labeled probe DNA molecules that competitively hybridized to compute the decisions^49^. Zhang and Seelig described an implementation of a linear classifier using DNA strand displacement^50^. Design of DNA circuits for supervised learning of a class of linear functions using buffered strand displacement reactions has been presented in^51^. Finally, Qian *et al*. demonstrated a complete artificial neural network, implemented experimentally using DNA strand displacement^52^.

In general, an artificial neural network consists of one or more layers where, in each layer, a neuron computes a weighted sum followed by a nonlinear activation (transfer) function. Typically the activation function corresponds to a sigmoid function. Prior work on molecular implementations of ANNs has considered either a hard-threshold^52^ or linear transfer function^50^ as the activation function.

This paper discusses the implementation of mathematical functions such as exponentials, trigonometric functions, the sigmoid function and a perceptron function with the limitation that the weighted sum of the inputs is scaled down by the dimension of the input vector.

In prior work on molecular computing, two types of *representation* for the input and output variables of chemical reaction networks (CRNs) have been considered:

1. The value of each variable corresponds to the concentration of a specific molecular type; this is referred to as a direct representation.
2. The value of each variable is represented by the difference between the concentrations of a pair of molecular types; this is referred to as a dual-rail representation^53^.

In recent work, we have proposed a new type of representation, referred to as a *fractional representation*^28^. Here a pair of molecular types is assigned to each variable, e.g., (*X*_0_, *X*_1_) for a variable *x*. The value of the variable is determined by the ratio of the concentrations for the assigned pair,

1 $$x=\frac{[{X}_{1}]}{[{X}_{0}]+[{X}_{1}]}$$

where [*X*_1_] and [*X*_0_] represent concentrations of molecules *X*_1_ and *X*_0_, respectively. Note that the value of *x* is confined to the unit interval, [0, 1]. With the values confined to the unit interval, we refer to the representations as a *unipolar* fractional encoding.

Variables with values in the range [−1, 1] can be represented by a slightly different encoding on the assigned pair (*X*_0_, *X*_1_), given by:

2 $$x=\frac{[{X}_{1}]-[{X}_{0}]}{[{X}_{0}]+[{X}_{1}]}\mathrm{.}$$

We refer to this representation as a *bipolar* fractional encoding.

The unipolar fractional coding and the connection that it makes between molecular computation and electronic stochastic logic design have been introduced in^28^. However, the extension of the idea for bipolar fractional coding and a systematic method for molecular implementation of complex functions using fractional coding has not been reported in prior work. The contributions of this paper are twofold. Firstly, molecular reactions are proposed to compute operations such as *ab*, 1 − *ab*, and *sa* + (1 − *s*)*b* using both the unipolar and bipolar fractional representations. These molecular circuits are, respectively, referred to as Mult, NMult, and MuX. Secondly, this paper demonstrates that unipolar and bipolar fractional coding approaches can be used to design CRNs for computing complex mathematical functions such as *e*^−*x*^, sin(*x*), and sigmoid (*x*). The proposed CRNs can readily be implemented using DNA strand displacement.

The fractional representation is inspired by a technique from digital electronic design, termed *stochastic logic*, in which values are represented by the probability of seeing 1’s in a stream of randomly generated 0’s and 1’s^54–59^. Research in the electronic realm has shown that a variety of complex functions can be computed with remarkably simple circuitry with this stochastic approach.

The main difference between^28^ and this paper lies in the approach proposed to design and synthesize computing CRNs. The approach in^28^ uses so-called control generating reactions and the transferring reactions that lead to reactions with *m* reactants for a polynomial of degree *m*. In contrast, this paper uses simple molecular units such as Mult and NMult described in the next section. Regardless of the complexity of the target functions, the molecular reactions designed by the new approach are only composed of simple reactions with two reactants and one product. These reactions are more suitable for DNA implementation. The molecular implementations presented in this paper are inspired by the stochastic implementations of functions presented in^60^.

The fractional encoding discussed in this paper is analogous to the stochastic representation. The concentrations of the *X*_0_ and *X*_1_ molecular types, correspond to the probability of seeing 0’s and 1’s, respectively, in the random streams. This paper demonstrates how stochastic electronic designs can be translated to molecular circuits.

One should notice that the bipolar fractional coding is just a representation of the value of a variable using two molecular types. This means that it is not required to actually calculate Equation (2). In other words, Equation (2) is our interpretation for the value of a variable and molecular reactions do not calculate this equation.

Section 1 introduces molecular reactions for the Mult and NMult units; these perform multiplication in the unipolar fractional representation. Section 2 presents an approach for mapping specific target functions to a cascade of Mult/NMult units. Section 3 introduces a molecular MUX unit that performs scaled addition, as well as Mult/NMult units for multiplication using the bipolar representation. Section 3 also presents an application: CRNs for implementing a single-layered neural network (also referred to as a perceptron). Section 4 discusses the DNA implementations of the proposed CRNs.

## CRNs for Multiplication Units

Based on the fractional coding discussed in the previous section, we propose two simple sets of CRNs for computing multiplication. We refer to these as Mult and NMult. These sets will serve as fundamental units in the construction of other desired functions in Section 2. Mult computes *c* = *a* × *b*, and NMult computes *c* = 1 − *a* × *b* where *a*,*b*, and *c* are in the unipolar fractional representation. The units are described below.

### Mult unit

**Figure 1:**
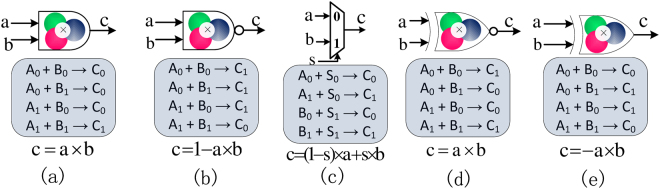
Basic molecular units. (**a**) The Multiplication unit, Mult. This unit calculates *c* = *a* × *b*, the multiplication of two input variables *a* and *b* in the unipolar fractional representation. (**b**) The NMult unit. This unit computes *c* = 1 − *a* × *b* in the unipolar fractional representation. (**c**) The MUX unit. This unit performs scaled addition. Here *a*,*b* and *c* can be in the unipolar or the bipolar representation, while *s* must be in in unipolar representation. (**d**) The bipolar Mult unit. This unit performs multiplication in the bipolar fractional representation. (**e**) The bipolar NMult unit. This unit computes *c* = −*a* × *b* in the bipolar fractional representation. Note the symbols that we will use to represent the different units are shown above the CRNs.

Consider the four reactions shown in Fig. 1(a). These compute *c* as the multiplication of two inputs *a* and *b*, all in unipolar fractional representation. So if $a=\frac{[{A}_{1}]}{[{A}_{0}]+[{A}_{1}]}$ and $b=\frac{[{B}_{1}]}{[{B}_{0}]+[{B}_{1}]}$ then $c=\frac{[{C}_{1}]}{[{C}_{0}]+[{C}_{1}]}=a\times b$. We prove this in Supplementary Section S.1, on the basis of both stochastic and ordinary dif ferential equations.

### NMult unit

If we switch *C*_0_ and *C*_1_ in the molecular reactions of the Mult unit, we obtain what we call an NMult unit which computes 1 − *a* × *b* in the unipolar fractional coding. Figure 1(b) shows the corresponding set of reactions. The proof that the NMult unit computes 1 − *a* × *b* is very similar to the proof for Mult unit. It can be obtained by switching *C*_0_ and *C*_1_ in the proof presented for Mult unit.

Note that the CRNs in Fig. 1 do not preserve the initial values of the input molecular types. The reactions can be modified such that the initial concentrations of either one or both of the input pairs, (*A*_0_, *A*_1_) and (*B*_0_, *B*_1_), are preserved. The details are presented in Section S.2 of the Supplementary Information.

Figure 1 shows three additional units. For some functions we use a CRN unit called MUX, shown in Fig. 1(c). To perform multiplication on the bipolar fractional coding, we use the CRN units shown in Fig. 1(d) and (e). All three CRN units are described in detail in Section 3 where we use them to compute the bipolar sigmoid function.

## Designing CRNs for Computing Functions

**Figure 2:**
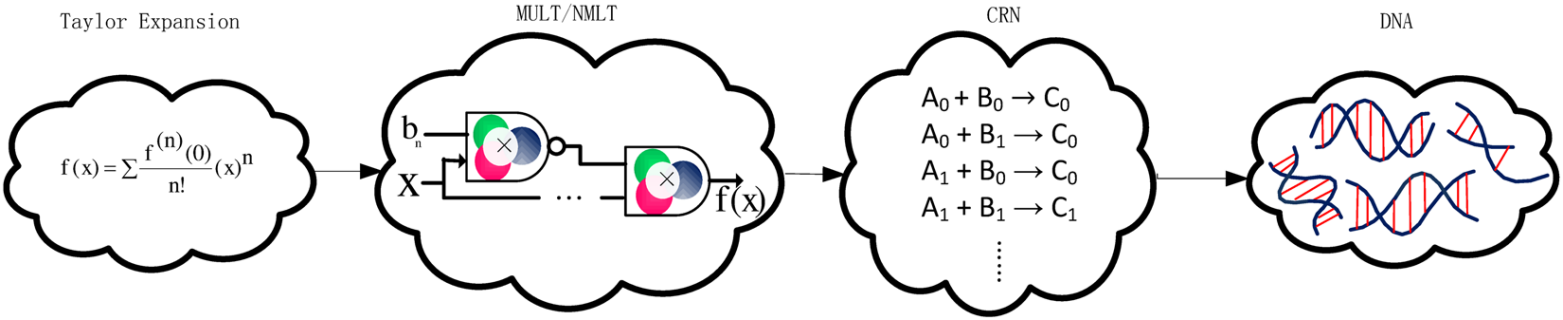
The proposed methodology. This figure shows the required steps for computing functions based on the proposed methodology. It starts with the approximation of the desired function as a polynomial using a series expansion method. The polynomial is then expressed in an equivalent form that only contains Mult and NMult units. The Mult and NMult units are mapped to their equivalent chemical reactions and finally the CRN is implemented by DNA strand-displacement reactions.

In this section we propose a framework for designing CRNs to compute different functions. Our method is illustrated in Fig. 2.

### Methodology

In the proposed methodology, the functions are approximated by truncating their Maclaurin series expansions. Note that other expansion methods such as Taylor series could also be used. The approximated polynomials are then mapped into equivalent forms that can be readily implemented using Mult and NMult units.

The Mult/NMult units are then mapped to CRNs. These are implemented by DNA strand-displacement reactions. We describe these steps using *f*(*x*) = *e*^−*x*^ as an example.

**Step 1- Approximate the function**

The Taylor series of any function *f*(*x*) that is infinitely differentiable at a point *a* corresponds to the power series

3 $$f(x)=\sum _{n=0}^{\infty }\frac{{f}^{(n)}(a)}{n!}{(x-a)}^{n}\mathrm{.}$$

If the Taylor series is centered at zero, i.e., *a* = 0, then the series is called a Maclaurin series. As an example for *f*(*x*) = *e*^−*x*^ the Maclaurin expansion is given by:

4 $${e}^{-x}=\sum _{n=0}^{\infty }\frac{{(-x)}^{n}}{n!}=1-x+\frac{{x}^{2}}{\mathrm{2!}}-\frac{{x}^{3}}{\mathrm{3!}}+\frac{{x}^{4}}{\mathrm{4!}}-\mathrm{....}$$

The series is truncated to a polynomial of degree *n*, in order to approximate the desired function. As an example if *n* = 5, i.e., the first six terms are retained, for *f*(*x*) = *e*^−*x*^ we obtain

5 $${e}^{-x}=1-x+\frac{{x}^{2}}{\mathrm{2!}}-\frac{{x}^{3}}{\mathrm{3!}}+\frac{{x}^{4}}{\mathrm{4!}}-\frac{{x}^{5}}{\mathrm{5!}}\mathrm{.}$$

**Step 2- Reformat the approximation and map it to** Mult **/** NMult **units**

As the second step, the approximating polynomials obtained in the first step are mapped into equivalent forms can be implemented using Mult and NMult units. The Mult and NMult units are analogous to AND and NAND gates in electronic design paradigm called stochastic processing. First developed by Poppelbaum^55^ and Gaines^56^ in the late 1960’s, stochastic processing implements logical computation on random bit streams. Numbers are encoded by the probability of obtaining a one versus a zero in stream of random bits.

In this work, the Mult and NMult units perform the same operation on molecular concentrations in the unipolar fractional encoding as AND and NAND gates do, respectively, in stochastic logic. Recent work in stochastic logic^60^ has shown that the form of polynomials that we generate in this step can be changed in a way that they can be mapped to a cascade of AND and NAND logic gates. The approach presented by Parhi and Liu uses the well known Horner’s rule in order to map polynomials with alternating positive and negative coefficients and decreasing magnitudes to AND and NAND gates^60^. This approach can be used for Maclaurin series of the functions *e*^−*x*^, sin(*x*), cos(*x*), log(1 + *x*), tanh(*x*), and sigmoid (*x*). Note that for the trigonometric functions, the operand *x* is in radians. We use the approach of Parhi and Liu^60^ to change the form of the desired approximating polynomials and then map them to a cascade of Mult and NMult units. We briefly describe this approach.

#### Horner’s rule

Consider a polynomial *P*(*x*) of degree *n* given in its power form as

6 $$P(x)={a}_{0}+{a}_{1}x+{a}_{2}{x}^{2}+{a}_{3}{x}^{3}+\mathrm{...}+{a}_{n}{x}^{n}\mathrm{.}$$

As described by Parhi and Liu^60^, Eq. (6) can be rewritten as

7 $$P(x)={b}_{0}\mathrm{(1}-{b}_{1}x\mathrm{(1}-{b}_{2}x\mathrm{(1}-{b}_{3}x\mathrm{...(1}-{b}_{n-1}x\mathrm{(1}-{b}_{n}x\mathrm{))))...)}$$

where *b*_0_ = *a*_0_ and ${b}_{i}=-\frac{{a}_{i}}{{a}_{i-1}}$ for *i* = 1, 2, ..., *n*. Provided 0 ≤ *b*_*i*_ ≤ 1 for *i* = 0, 1, ...., *n*, this representation can be easily mapped to a regular cascade of molecular Mult and NMult units as described by Parhi and Liu^60^.

In order to guarantee 0 ≤ *b*_*i*_ ≤ 1 the following requirements must be satisfied. Firstly, the coefficients of the original polynomial, i.e., the *a*_*i*_’s, should be alternatively positive and negative. Secondly, the absolute values for all the coefficients, i.e., the *a*_*i*_’s, should be less than one and decrease as the terms’ orders increase. There exist several polynomials that satisfy these requirements. For example Maclaurin series expansion of *e*^−*x*^, sin(*x*), cos(*x*), log(1 + *x*), tanh(*x*), and sigmoid (*x*), listed in equations (41) to (46) of the Supplementary Information, meet these requirements and can be represented using Equation (7).

Consider the following example. If we apply the Horner’s rule for the fifth order Maclaurin series of *f*(*x*) = *e*^−*x*^, shown in (5), we obtain

8 $${e}^{-x}=1-x(1-\frac{x}{2}(1-\frac{x}{3}(1-\frac{x}{4}(1-\frac{x}{5}))))\mathrm{.}$$

**Figure 3:**
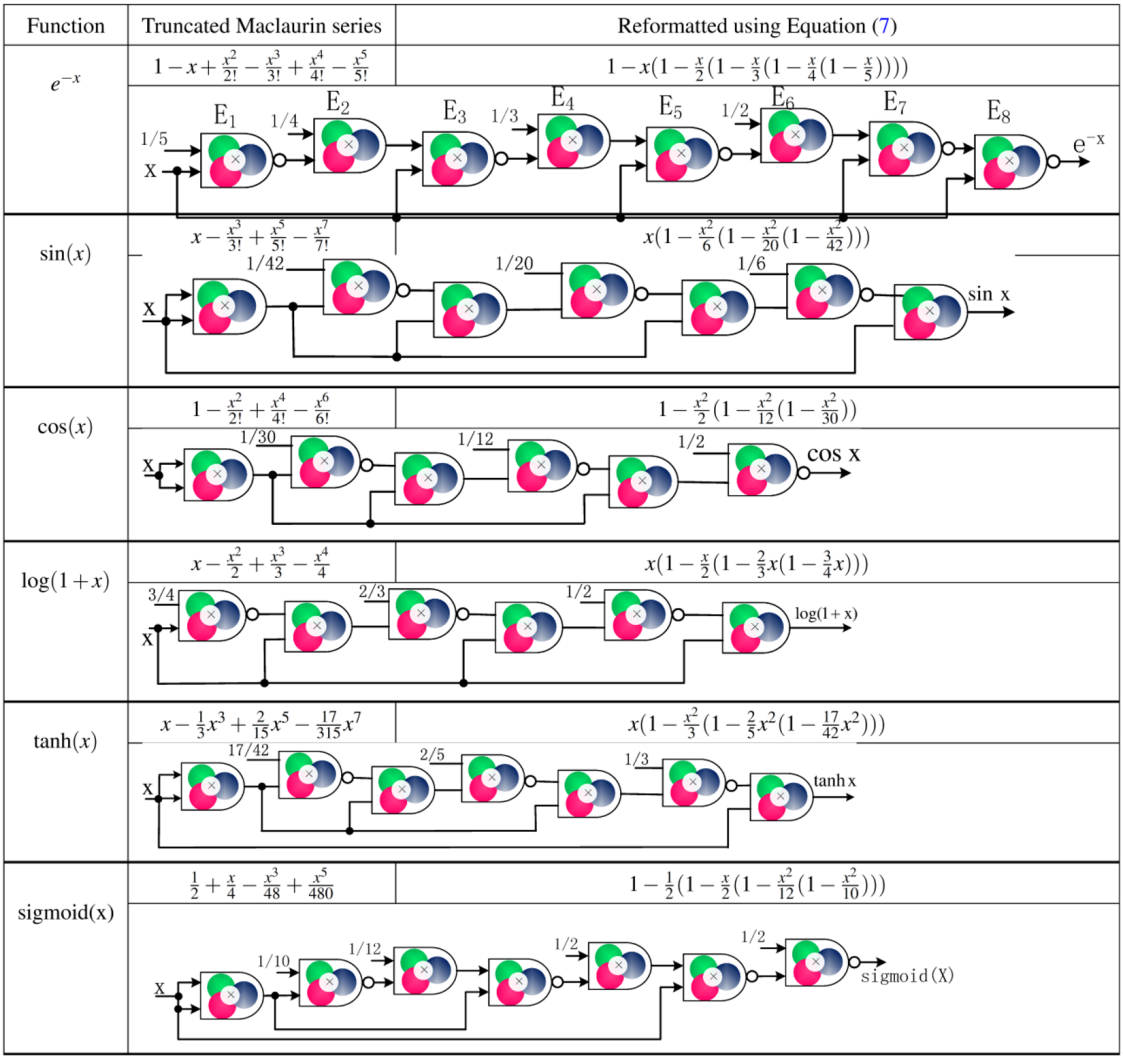
Examples of molecular circuits for mathematical functions. Truncated Maclaurin series, reformatted Maclaurin series using Horner’s rule, and Mult/NMult structure for functions in equations (41)–(46) of the Supplementary Information.

Equation (8) can be implemented using Mult and NMult units as shown in Fig. 3.

Elements *E*_*i*_ of the structure shown in Fig. 3 compute intermediate outputs *t*_*i*_ in order to progressively compute the *e*^−*x*^ function using Equation (8). What follows is the computation for each element:

$$\begin{array}{c}{{\rm{E}}}_{1}:\,{t}_{1}=(1-\frac{x}{5})\,\,\,{{\rm{E}}}_{2}:\,{t}_{2}=\frac{1}{4}{t}_{1}\,\,\,\,{{\rm{E}}}_{3}:\,{t}_{3}=(1-\frac{x}{4}{t}_{1})\\ {{\rm{E}}}_{4}:\,{t}_{4}=\frac{1}{3}{t}_{3}\,\,\,\,\,\,{{\rm{E}}}_{5}:\,{t}_{5}=(1-\frac{x}{3}{t}_{3})\,\,{{\rm{E}}}_{6}:\,{t}_{6}=\frac{1}{2}{t}_{5}\\ {{\rm{E}}}_{7}:\,{t}_{7}=(1-\frac{x}{2}{t}_{5})\,\,{{\rm{E}}}_{8}:\,f(x)=1-x{t}_{7}={e}^{-x}.\end{array}$$

**Figure 4:**
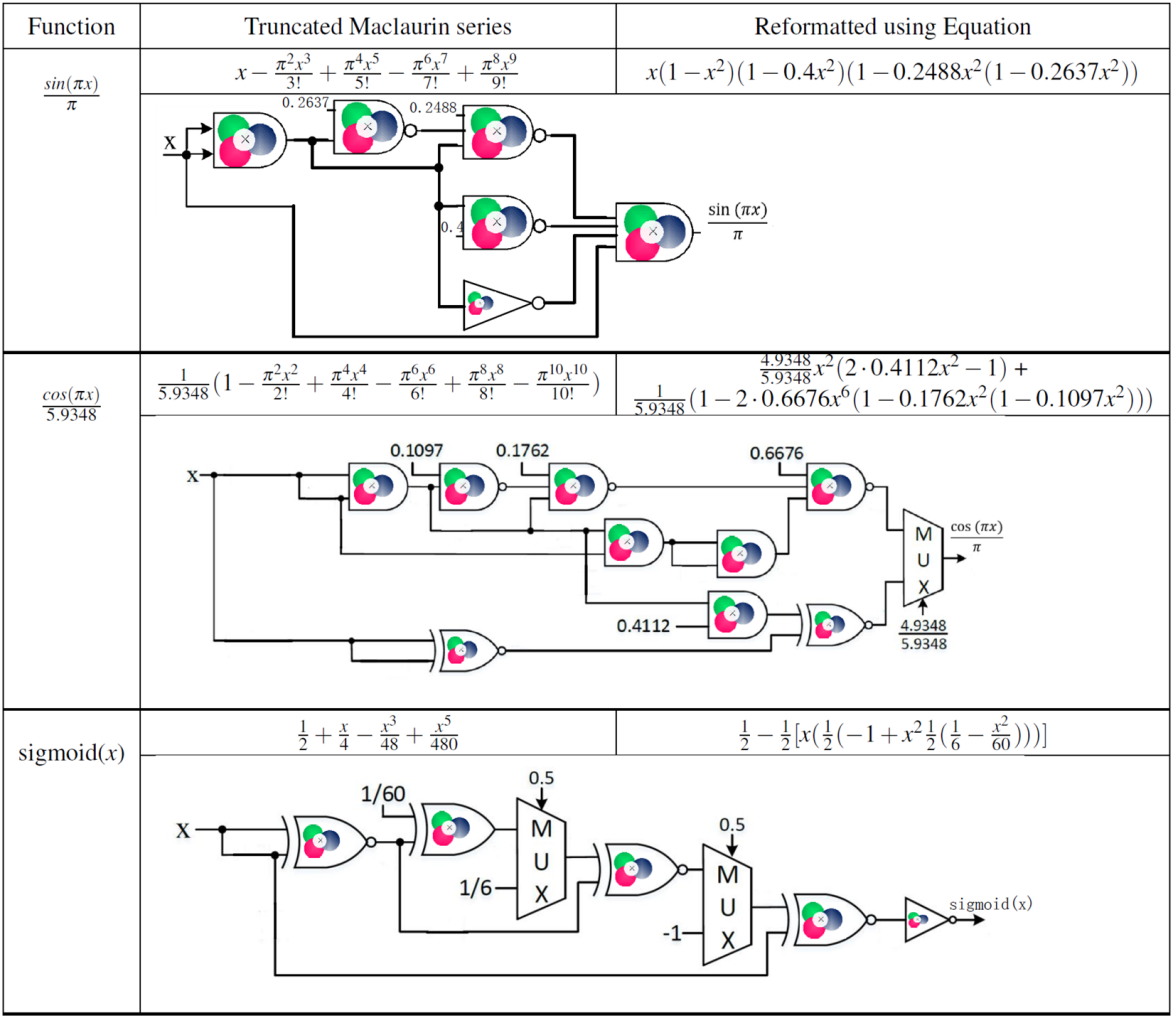
Examples of molecular circuits for mathematical functions with inputs covering entire range. Truncated Maclaurin series, reformatted Maclaurin series using Horner’s rule, Mult/NMult and MUX structure for functions in equations (47), (48) and (46) of the Supplementary Information. The output of the cosine function and the input of the bipolar sigmoid are in bipolar representation.

Figure 3 summarizes the truncated Maclaurin series, reformatted Maclaurin series using Horner’s rule, and Mult/NMult structure for several other desired functions where the input and output are in unipolar representation. Figure 4 presents Mult/NMult structure for stochastic logic implementations of half-period of $\frac{sin(\pi x)}{\pi }$ and $\frac{cos(\pi x)}{5.9348}$ as presented in Parhi and Liu^60^ and described by equations (9) and (10). Note that in the scaled cosine computation, the input is in unipolar representation while the output is in bipolar representation and can represent negative values. This is referred to as hybrid representation^61^.

9 $$\frac{sin(\pi x)}{\pi }=x\mathrm{(1}-{x}^{2}\mathrm{)(1}-0.4{x}^{2})(1-0.2488{x}^{2}\mathrm{(1}-0.2637{x}^{2}))\mathrm{.}$$

10 $$\begin{array}{rcl}\frac{cos(\pi x)}{5.9348} & = & \frac{4.9348}{5.9348}{x}^{2}\mathrm{(2}\cdot 0.4112{x}^{2}-\mathrm{1)}\\  &  & +\frac{1}{5.9348}\mathrm{(1}-2\cdot 0.6676{x}^{6}\mathrm{(1}-0.1762{x}^{2}\mathrm{(1}-0.1097{x}^{2}\mathrm{))).}\end{array}$$

**Step 3- Synthesize the Chemical Reactions**

To build the CRN for computing the desired function, the next step is to synthesize the related chemical reactions for each element used in the Mult/NMult structure. Depending on the unit type, either the set of reactions presented in Fig. 1(a–c) is used. After designing the CRNs, the final step is to map them to DNA reactions as described in Section 4. Note that Mult/NMult units with more than two inputs are built by cascading two-input Mult/NMult units.

## Molecular Perceptron

**Figure 5:**
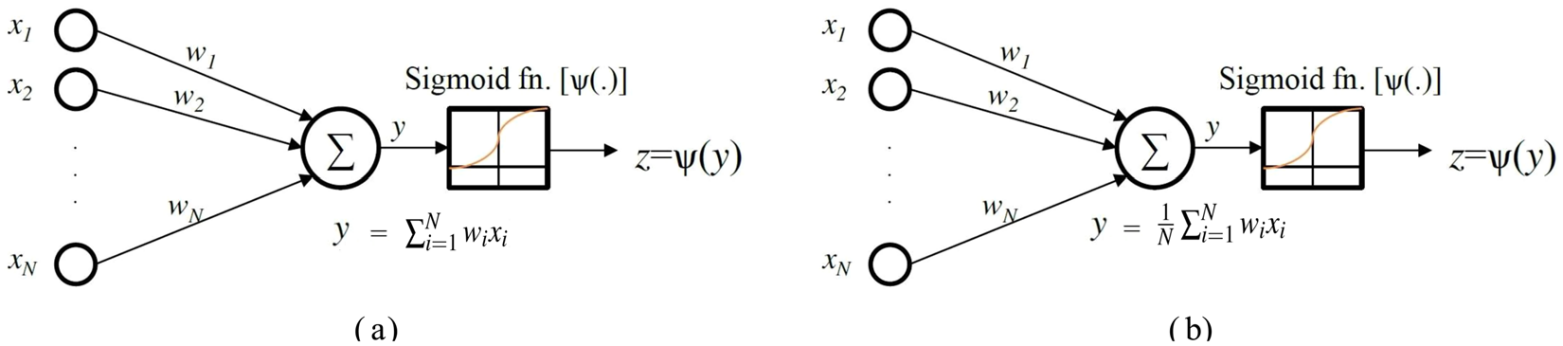
Molecular Perceptron. (**a**) A standard perceptron that computes sigmoid $({\sum }_{i=1}^{N}{w}_{i}{x}_{i})$, (**b**) A molecular perceptron that computes sigmoid $(\frac{1}{N}{\sum }_{i\mathrm{=1}}^{N}{w}_{i}{x}_{i})$.

This section describes implementation of a single-layered neural network, also called a perceptron, by molecular reactions. As shown in Fig. 5(a), the system first computes the inner product of a binary input vector and a coefficient vector as $y={\sum }_{i\mathrm{=1}}^{N}{w}_{i}{x}_{i}+{w}_{0}$; then it uses the sigmoid function to compute the final output *z* as *z* = sigmoid (*y*). The stochastic sigmoid circuit shown in Fig. 5(b) was presented in^60^ the reader is referred to^60^ for details of the derivation. This performs a soft decision of whether the output should be close to 0 or 1. For the perceptron system that we implement, the inputs are binary, that is to say either *x*_*i*_ = 0 or *x*_*i*_ = 1, and the coefficients, i.e., *w*_*i*_’*s*, are between −1 and 1. All multiply-add operations are implemented using bipolar Mult units. Since the input of the sigmoid function is between −1 and 1, we implement the sigmoid function using a bipolar fractional coding.

Note that prior biomolecular implementations of artificial neural networks (ANNs) have considered either hard limit or linear activation functions^50,52^. No prior publication has considered molecular ANNs using a sigmoid activation function. In this section we describe the implementation of the bipolar MUX unit and the bipolar Mult and NMult units.

### MUX unit

The MUX unit, shown in Fig. 1(c), computes *c* as the weighted addition of two inputs *a* and *b* as *c* = *a* × (1 − *s*) + *b* × *s*, where 0 ≤ *s* ≤ 1. Here *a*, *b*, and *c* can be in either the unipolar or the bipolar fractional representation while the weight *s* must be in the unipolar representation. The set of four reactions in Fig. 1(c) describes the CRN for a MUX unit for both unipolar and bipolar fractional codings. Mass-action kinetic equations for both unipolar and bipolar fractional coding are discussed in Supplementary Information Section S.4.

### Bipolar Mult unit

The bipolar Mult unit, shown in Fig. 1(d), computes *c* as the multiplication of two inputs *a* and *b*, where *a*, *b* and *c* are represented in bipolar fractional representation. So if $a=\frac{[{A}_{1}]-[{A}_{0}]}{[{A}_{0}]+[{A}_{1}]}$ and $b=\frac{[{B}_{1}]-[{B}_{0}]}{[{B}_{0}]+[{B}_{1}]}$ then $c=\frac{[{C}_{1}]-[{C}_{0}]}{[{C}_{0}]+[{C}_{1}]}=a\times b$. The set of four reactions in Fig. 1(d) represents the CRN for a multiplication unit in the bipolar fractional coding. In Supplementary Information Section S.3 we prove that these reactions compute *c* = *a* × *b*.

### Bipolar NMult unit

Analogous to the way that we obtained NMult from Mult unit in the unipolar fractional coding, if we switch *C*_0_ and *C*_1_ in the reactions of the bipolar Mult unit, we obtain the bipolar NMult unit which computes −*a* × *b*. Figure 1(e) gives the corresponding set of reactions. Similar to the method we used for Mult unit, it is easy to show that the reactions listed in Fig. 1(e) compute *c* = −*a* × *b* in the bipolar fractional coding.

The proof is very similar to the bipolar Mult unit. Indeed, for bipolar NMult we just switch *C*_0_ and *C*_1_ meaning that in the proof for bipolar Mult instead of *C*_1_ − *C*_0_ in the numerator we have *C*_0_ − *C*_1_. This leads to having *c* = −*ab* instead of *c* = *ab*.

### Hybrid sigmoid function and Perceptron with Binary Inputs

The bipolar fractional representation can be used to implement the sigmoid function, presented in Section 2.1.1 for the unipolar fractional representation. Therefore, the function can be computed for inputs between −1 and 1, i.e., −1 ≤ *x* ≤ 1. The output of this function, however, is still in the unit interval [0, 1] and can be represented by a unipolar fractional representation. In fact, for *x* ∈ [−1, 1] the corresponding output range is [0.2689, 0.7311]. In Parhi and Liu^60^, it is shown that the sigmoid function using hybrid format, i.e., for bipolar input and unipolar output can be implemented by electronic stochastic logic circuits, namely, XOR and XNOR gates and multiplexers. These electronic circuits perform multiplication and weighted addition for stochastic bit streams analogous to the same operations that bipolar Mult, NMult, and MUX units in Fig. 1 perform for CRNs. Accordingly, we map the circuit to the cascade of proposed molecular units as shown in Fig. 5(b). The inner product can be implemented by *N* bipolar Mult units having the same output. Details for the molecular implementation of the inner product are described in Section S.5 of the Supplementary Information.

**Figure 6:**
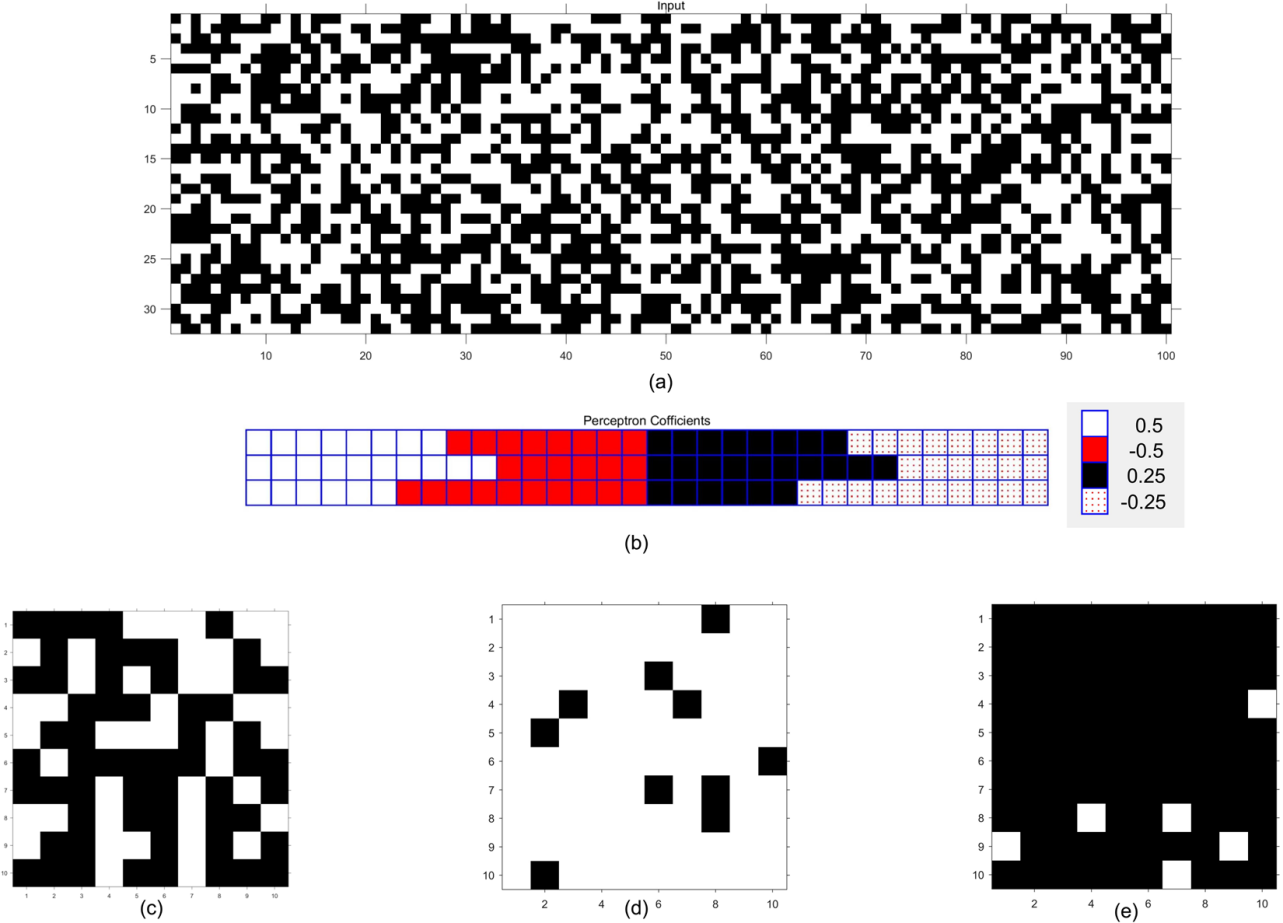
Inputs, weights and outputs of three perceptrons, denoted A, B and C. (**a**) Inputs to the perceptron: each column of the 32×100 matrix illustrates an input vector containing 32 binary inputs. Each white square corresponds to a 1 and each black square corresponds to a 0. (**b**) Weights: The weights for the three perceptrons are illustrated. These weights are divided into 4 parts and correspond to 1/2, −1/2, 1/4 and −1/4 from left to right. (**c**) Binary outputs of Perceptron A containing 58 1’s and 42 0’s. (**d**) Binary outputs of Perceptron B containing 90 1’s and 10 0’s. (**e**) Binary outputs of Perceptron C containing 6 1’s and 94 0’s.

By cascading the inner product part and the sigmoid function, we can implement molecular perceptrons with binary inputs as shown in Fig. 5. Although the inner product in the standard perceptron shown in Fig. 5(a) computes ${\sum }_{i\mathrm{=1}}^{N}{w}_{i}{x}_{i}$, the molecular inner product in Fig. 5(b) computes $\frac{1}{N}{\sum }_{i\mathrm{=1}}^{N}{w}_{i}{x}_{i}$. We map this molecular circuit to DNA strand-displacement reactions and simulate it for *N* = 32 using 32 coefficients. Three perceptrons are simulated. The 32 binary inputs are selected at random such that each bit is equally likely to be 1 or 0. It is important to note that the inputs are not constrained to be binary in the proposed methodology, but are constrained to lie between −1 and 1. For each perceptron, the same 100 input vectors are simulated. The input vectors are illustrated in Fig. 6(a) where the 100 columns correspond to 100 input vectors, and each column contains 32 binary values chosen at random with equal probability. The corresponding binary matrix representing the 100 input vectors is also shown in Figure S.7.1 in the Supplementary Information Section S.7. The weights of perceptrons are chosen from the set 1/2, −1/2, 1/4, and −1/4. These weights for the 3 perceptrons, denoted A, B and C, are illustrated in Fig. 6(b), and are also listed in Supplementary Section S.7. In Perceptron A, each weight occurs 8 times. In Perceptron B, the weights 1/2, −1/2, 1/4 and −1/4, occur with frequencies 10, 6, 10 and 6, respectively, In Perceptron C, the weights 1/2, −1/2, 1/4, and −1/4 occur with frequencies 6, 10, 6, and 10, respectively. In a perceptron, let the presence or absence of the input molecules be denoted by 1 or 0, and the coefficients describe the weights associated with each input, and each weighted molecule either activates or inhibits the perceptron *state* depending on whether it is positive or negative. Then Perceptron B has more molecules that activate the state whereas Perceptron C has more molecules that inhibit the state, whereas Perceptron A has equal number of molecules that either activate or inhibit the state. For equally likely binary inputs, the probabilities of the weighted sum for the Perceptrons A, B, and C, respectively, correspond to 0, 1.5 and −1.5. The expected sigmoid values for the three perceptrons correspond to 0.5, 0.8175, and 0.1825, respectively. Each perceptron output is classified as 1 or 0 using a threshold of 0.5. If very large number of random input vectors are simulated, we would expect the percent of input vectors classified as 1 in these three perceptrons to be 50%, 81.75% and 18.25%, respectively. For the 100 input vectors, the classification results for the three perceptrons are illustrated in Fig. 6(c–e). The number of 1’s in these perceptrons correspond to 58, 90 and 6, respectively. All three molecular perceptrons achieve classification accuracy of 100%.

**Figure 7:**
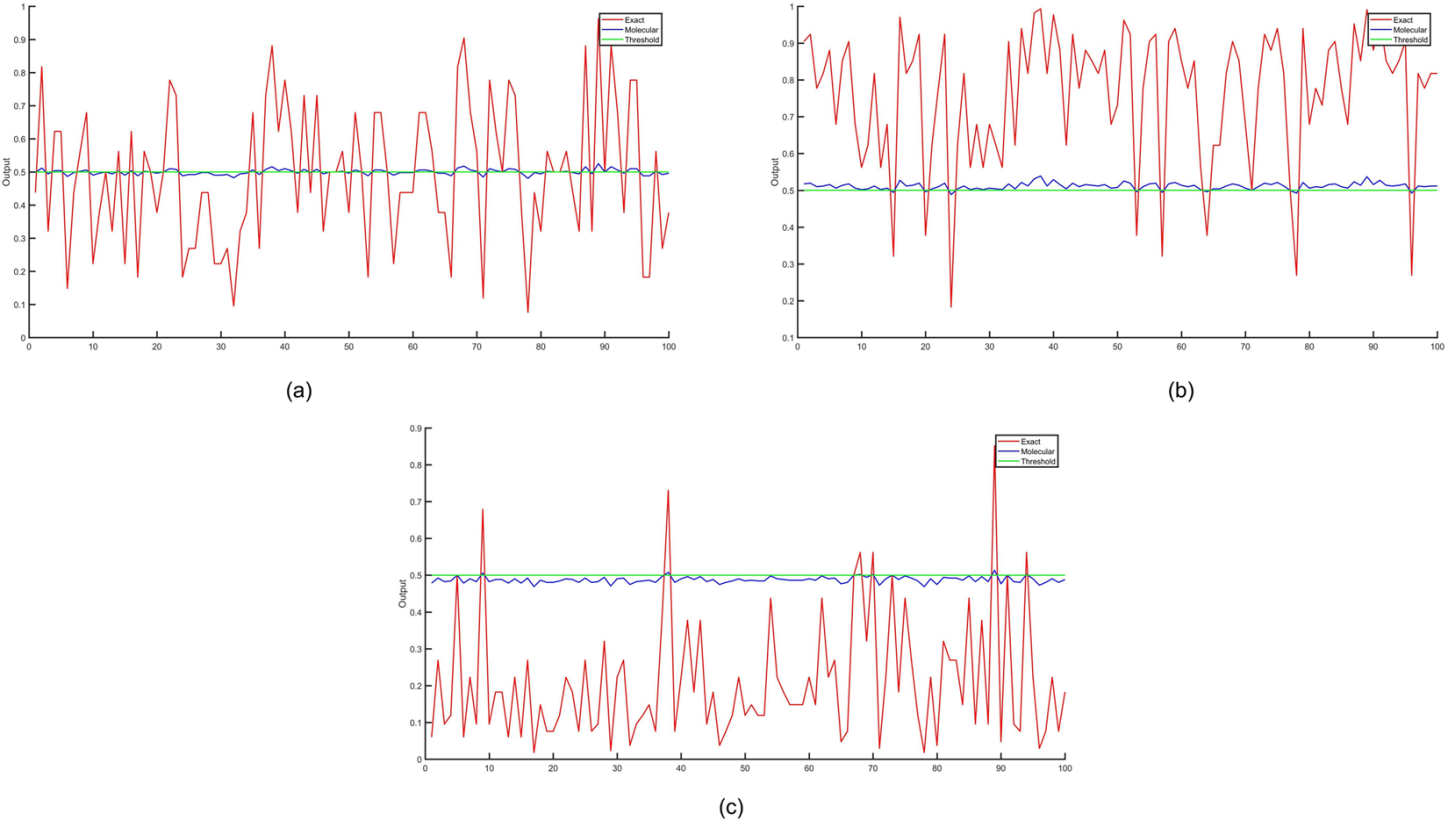
Exact perceptron outputs that represent sigmoid of the weighted sum of the inputs and the molecular perceptron outputs that compute sigmoid of the weighted sum scaled by a factor 1/32 for the 100 input vectors for: (**a**) Perceptron A, (**b**) Perceptron B, (**c**) Perceptron C. The *x* axis corresponds to input vector number.

**Figure 8:**
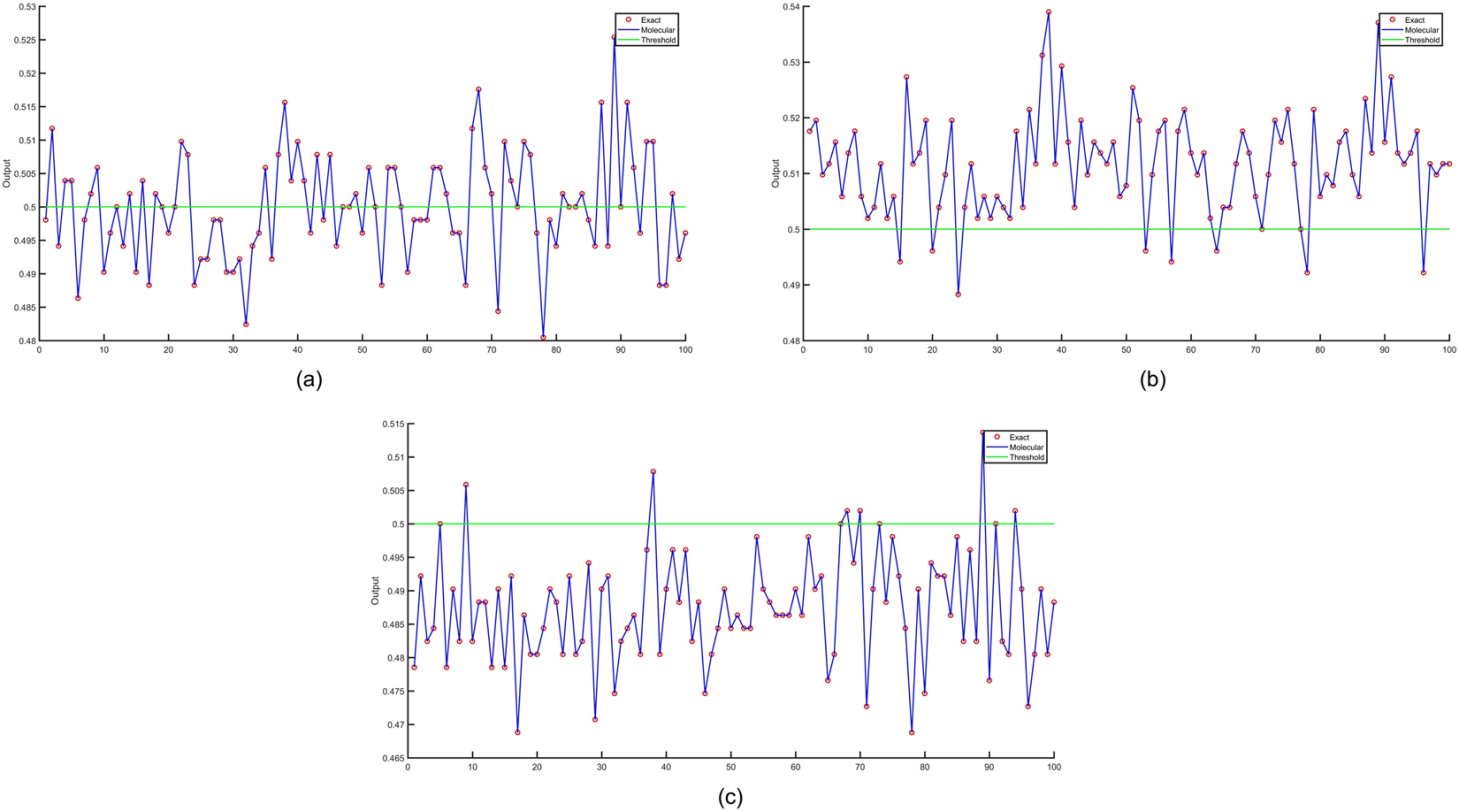
Exact and molecular perceptron outputs with weighted sum of the inputs scaled by 1/32 for 100 input vectors for: (**a**) Perceptron A, (**b**) Perceptron B, (**c**) Perceptron C. The *x* axis corresponds to input vector number.

The simulation results in Fig. 7(a–c) illustrate the exact sigmoid values of the weighted sum of the inputs and the outputs of the molecular perceptrons that compute sigmoid of the weighted sum of the inputs scaled down by the dimension of the input vector, i.e., 32, for the Perceptrons A, B, and C, respectively. The horizontal axis in Fig. 7 represents the index of the input vector and the vertical axis shows the exact sigmoid value and the molecular sigmoid value. Although the molecular CRN outputs do not perfectly match with actual values, if we consider 0.5 as the threshold for a binary decision, the molecular perceptron classification results and the actual perceptron classifier results are the same for all 100 input vectors. Since the molecular inner product computes $y=\frac{1}{N}{\sum }_{i\mathrm{=1}}^{N}{w}_{i}{x}_{i}$ instead of $y={\sum }_{i\mathrm{=1}}^{N}{w}_{i}{x}_{i}$, the amplitude for the computed output is not same as the exact value. Note that *x*_*i*_ and *w*_*i*_, respectively, represent the binary value of the *i*^th^ input and its associated weight. Figure 8 shows the exact and molecular outputs of the three perceptrons that compute sigmoid of the scaled versions of the weighted inputs for the 100 input vectors. The next section describes DNA implementations of the proposed CRNs.

## DNA Implementation

Constructs in the previous sections were presented in terms of abstract CRNs. In this section, we translate our Mult/NMult circuits to DNA strand displacement (DSD) reactions. The idea of DSD reactions based on toehold mediation was first introduced by Yurke *et al*. for the construction of DNA tweezers^2^. A general method for translating CRNs to DSD reactions was proposed by Soloveichik *et al*.^6^ and is illustrated in Supplementary Information Section S.8 and Figure S.8.1. That work proved that DSD reactions can closely emulate the mass-action kinetics of any CRN.

Recently Chen *et al*. showed that bimolecular reactions, such as *A* + *B* → *C*, can be implemented by linear, double-stranded DNA complexes that are compatible with natural DNA^32^. We note that our computational units are all constructed from bimolecular reactions and so these could be implemented using the framework proposed by Chen *et al*.^32^.

**Figure 9:**
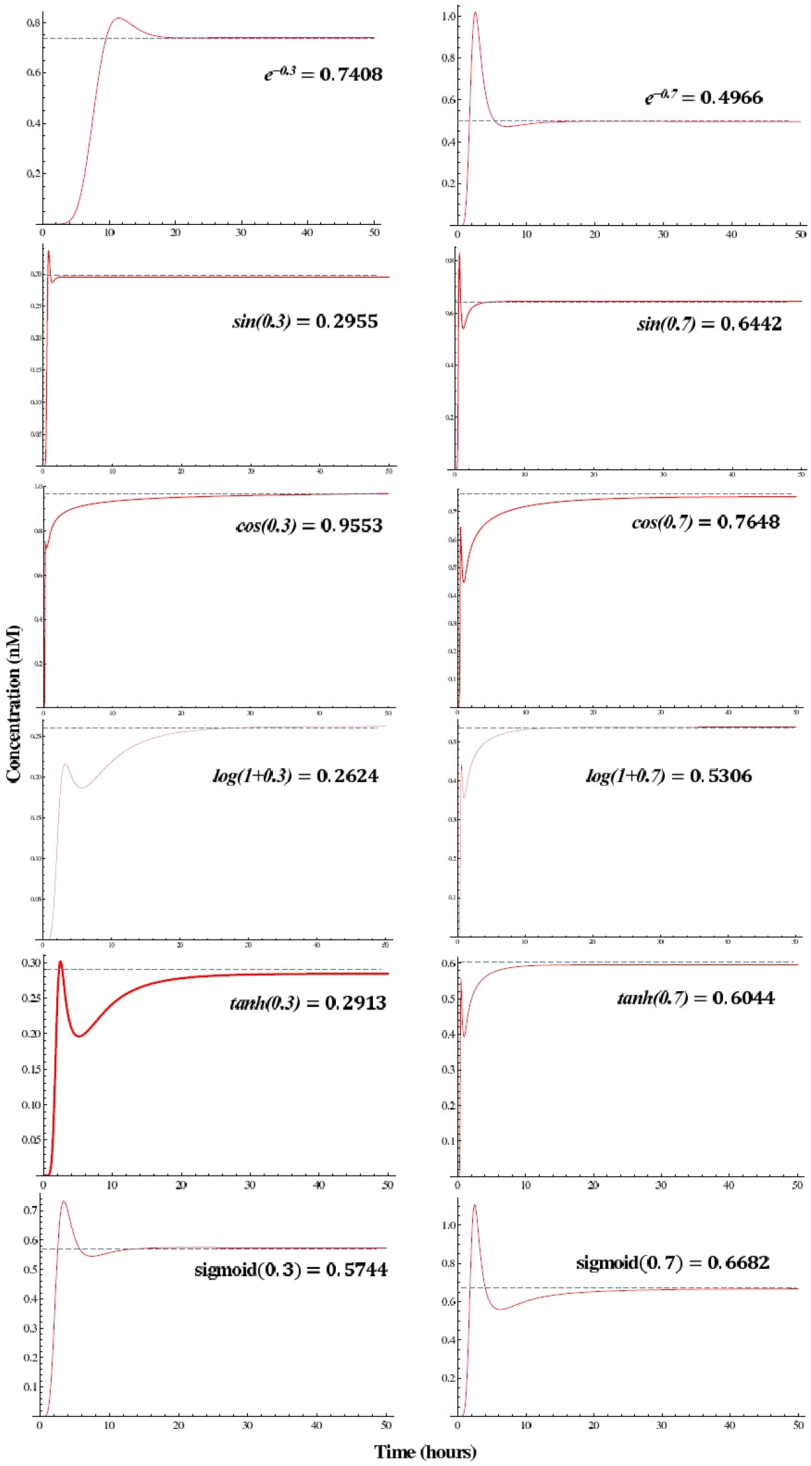
DNA simulation results. The DNA reaction kinetics for the computation of *e*^−*x*^, sin(*x*), cos(*x*), log(1 + *x*), tanh(*x*), and sigmoid(*x*) for *x* = 0.3, and *x* = 0.7. Each row pertains to one function. The details for the DNA implementation are listed in Supplementary Information Section S.8.

**Figure 10:**
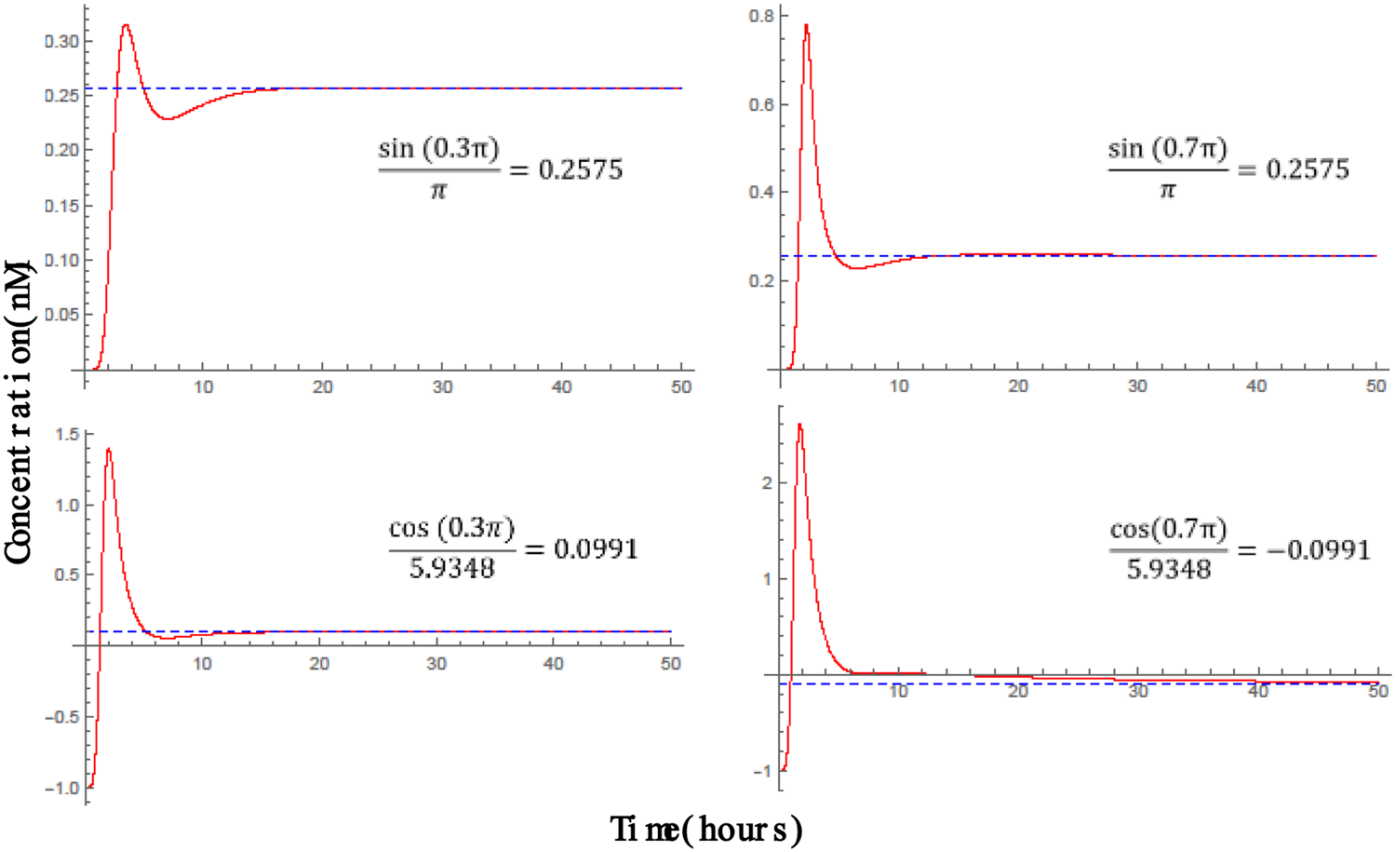
DNA simulation results. The DNA reaction kinetics for the computation of $\frac{sin(\pi x)}{\pi }$, and $\frac{cos(\pi x)}{5.9348}$ for *x* = 0.3, and *x* = 0.7. For the cosine function, the simulation shows $\frac{[{Y}_{1}]-[{Y}_{0}]}{[{Y}_{0}]+[{Y}_{1}]}$, where *Y*_0_ and *Y*_1_ represent the output in bipolar fractional coding.

Using the software tool provided by Erik Winfree’s group in Caltech^6^ we simulate the reactions using DSD. Figures 9 and 10 show the simulation results for the functions at *x* = 0.3 and *x* = 0.7. Table 1 presents simulation data highlighting the accuracy of the proposed method. It lists computed values for functions at eleven equally separated points in the interval [0,1]. For each function, the computed result is reported 50 hours after the simulation starts. The table also lists the *mean square error* (MSE) at the eleven points. The error may be due to several factors: the approximation of the function with a truncated series expansion; the emulation of the related CRNs by DSD reactions; and the limited simulation time (of 50 hours for DSD reactions). As the results show, the error is less than 1 × 10^−3^. For a visual comparison, Figure S.8.2 of the Supplementary Information illustrates the exact values of the functions together with their computed values.

**Table 1:** Computed values of functions with the proposed CRNs compared to their exact values.

Function		x = 0	x = 0.1	x = 0.2	x = 0.3	x = 0.4	x = 0.5	x = 0.6	x = 0.7	x = 0.8	x = 0.9	x = 1	MSE
*e* ^− *x*^	computed	0.9568	0.8770	0.7975	0.7228	0.6609	0.5951	0.5295	0.4772	0.4300	0.3872	0.3482	5.02 × 10^−4^
	exact	1	0.9048	0.8187	0.7408	0.6703	0.6065	0.5488	0.4966	0.4493	0.4066	0.3679	
sin(*x*)	computed	0	0.1045	0.2062	0.3043	0.3970	0.4833	0.5570	0.6261	0.6844	0.7460	0.7967	4.63×10^−4^
	exact	0	0.0998	0.1986	0.2955	0.3894	0.4794	0.5646	0.64421	0.7173	0.7833	0.8414	
cos(*x*)	computed	0.9728	0.9757	0.9641	0.9407	0.9129	0.8671	0.8071	0.7461	0.6778	0.6029	0.5221	3.16 × 10^−4^
	exact	1	0.9950	0.9800	0.9553	0.9210	0.8775	0.8253	0.7648	0.6967	0.6216	0.5403	
log(1 + *x*)	computed	0.0090	0.0985	0.1868	0.2675	0.3410	0.4075	0.4660	0.5212	0.5707	0.6217	0.6699	1.8 × 10^−4^
	exact	0	0.0953	0.1823	0.2623	0.3364	0.4054	0.4700	0.5306	0.5877	0.6418	0.6931	
tanh(*x*)	computed	0	0.0935	0.1883	0.2823	0.3701	0.4574	0.5277	0.5826	0.6246	0.6682	0.7038	7.35 × 10^−4^
	exact	0	0.0996	0.1973	0.2913	0.3799	0.4621	0.5370	0.6043	0.6640	0.7162	0.7615	
sigmoid(x)	computed	0.5196	0.5453	0.5657	0.5878	0.6068	0.6212	0.6366	0.6570	0.6721	0.6906	0.7084	2.5 × 10^−4^
	exact	0.5000	0.5250	0.5498	0.5744	0.5987	0.6225	0.6457	0.6682	0.6900	0.7109	0.7311	
$\frac{sin(\pi x)}{\pi }$	computed	0	0.0984	0.1871	0.2574	0.3023	0.3176	0.3016	0.2565	0.1931	0.1329	0.0899	8.48 × 10^−4^
	exact	0	0.0984	0.1871	0.2576	0.3027	0.3183	0.3027	0.2575	0.1871	0.0984	0	
$\frac{cos(\pi x)}{5.9348}$	computed	0.1685	0.1602	0.1363	0.0991	0.0527	0.0030	−0.0429	−0.07729	−0.0921	−0.0852	−0.0673	1.67 × 10^−3^
	exact	0.1685	0.1603	0.1363	0.0990	0.0521	0	−0.0521	−0.0990	−0.1363	−0.1603	−0.1685	

Table 2 lists the classification accuracy of the three perceptrons simulated using DSD with results collected after 50 hours of simulations.The table also lists the mean square error values for the three perceptrons for both molecular reactions and DNA strand displacement reactions. The mean square error, MSE, is defined as:

$$MSE=\frac{1}{100}\sum _{j=1}^{100}{|y(j)-\widehat{y}(j)|}^{2}$$

where $y(j)=sigmoid(\frac{1}{N}{\sum }_{i\mathrm{=1}}^{N}{w}_{i}{x}_{i}[j])$ and $\widehat{y}(j)$ is the computed value of *y*(*j*) from molecular or DNA simulation, *x*_*i*_[*j*] represents the *i*^th^ bit position of input vector *j*, and *w*_*i*_ represents the *i*^th^ weight. The mean square error values for molecular and DNA simulations are small as the dynamic range of the sigmoid function with scaled weighted sum of binary inputs is small. For example, *sigmoid*(1.5/32) and *sigmoid*(−1.5/32), respectively, correspond to 0.5117 and 0.4882. Although the DNA implementation of the perceptron achieves 100% classification accuracy in simulation, we caution that in an actual experiment the DNA perceptron may not achieve perfect classification accuracy.

**Table 2:** Classification accuracy and mean sqaure error values for the three perceptrons with weighted input values scaled by factor 1/32 for molecular reactions and DNA strand displacement reactions.

Perceptron	TP	TN	FP	FN	Mean Square Error	
					Molecular	DNA
A	58	42	0	0	2.0198 × 10^−11^	3.7670 × 10^−7^
B	90	10	0	0	1.2301 × 10^−10^	1.0357 × 10^−6^
C	6	94	0	0	3.9050 × 10^−12^	9.1999 × 10^−8^

## Conclusion

Although there have been numerous examples of CRNs for computing specific functions presented in the literature, as yet there has been no *systematic* way to design molecular systems to compute mathematical functions. This paper presents a systematic methodology for designing CRNs to implement complex mathematical functions robustly. The proposed method is unique in that it relies exclusively on bimolecular reactions, with no requirements on the reaction rates. According to the work of Chen *et al*., bimolecular reactions are compatible with natural DNA^32^. This means that, the computational elements we propose here could potentially be used for *in vivo* applications. A key contribution of this paper is the ability to map any stochastic logic circuit to a molecular circuit based on fractional coding. Numerous prior papers have demonstrated stochastic logic implementations of digital filters, error control coders such as low-density parity check codes and polar codes. The proposed molecular logic gates can be used to design molecular digital filters and molecular error control coders in a straightforward manner.

This paper builds on our prior work. The computation of polynomials was presented in Salehi *et al*.^28^. In that paper we showed how arbitrary polynomials can be mapped to a CRN. Although that method could be used to compute truncated Maclaurin series of desired functions, it uses a rather complex set of chemical reactions with *m* reactants and at least *m* + 1 products, with *m*≥2, for polynomials of degree *m*. Implementing reactions with more than two reactants may be biologically infeasible, since this entails large complexes. In contrast, the methodology proposed in this paper requires only bimolecular reactions and so is readily implementable.

Although molecular and DNA implementations of several mathematical functions using fractional coding have been demonstrated, the proposed method suffers from numerous limitations. Use of fractional coding, inspired by stochastic logic^62–66^, requires molecules to be bounded between −1 and 1. Thus, complete dynamic range of a function cannot be computed by the proposed method. For example, the proposed method can only compute scaled sine and cosine values. The molecular perceptron cannot compute the sigmoid value of the weighted sum of the binary inputs. This is an inherent limitation of the proposed method as the sigmoid function processes a scaled version of the weighted inputs (scaled down by the dimension of the input vector). Furthermore, the weight values are constrained to lie between −1 and 1. Molecular implementations of general perceptrons with arbitrary weights remains a topic for future research. In addition, further research needs to be directed towards molecular implementations of perceptrons used in inference applications as opposed to binary classification applications.

## Electronic supplementary material

Supplementary Information
