# Supplementary material for: Computing Mathematical Functions using DNA via Fractional Coding
Source: Sci Rep. 2018 May 29;8:8312. doi: 10.1038/s41598-018-26709-6 (PMC5974329; doi:10.1038/s41598-018-26709-6)
Supplement: Supplementary file 1 — Supplementary Information [file 41598_2018_26709_MOESM1_ESM.pdf]

Supplementary Information
Computing Mathematical Functions using DNA via Fractional Coding

Sayed Ahmad Salehi, Xingyi Liu, Marc D. Riedel, and Keshab K. Parhi

April 6, 2018

S.1 Proof for Unipolar Mult Unit

In this section, using both stochastic modeling and mass-action kinetics, we prove that the Mult unit computes product of its inputs in unipolar fractional representation.

S.1.1 Stochastic model:

Based on the stochastic modeling of molecular behavior, we describe how chemical reactions in Fig. 1(a) of the main text compute the multiplication operation. The reactions are represented in (1) as $R1$ to $R4$.

$$\begin{aligned} R1: &\quad A_0 + B_0 \rightarrow C_0 \\ R2: &\quad A_0 + B_1 \rightarrow C_0 \\ R3: &\quad A_1 + B_0 \rightarrow C_0 \\ R4: &\quad A_1 + B_1 \rightarrow C_1 \end{aligned} \tag{1}$$

In the stochastic model the concentrations of molecules are considered as discrete quantities and they are used to analyze the behavior of molecular reactions. The probabilities of firing each one of the four reactions listed in (1) are shown by four equations in (2), respectively.

$$P(R1) = \frac{\binom{A_0}{1}\binom{B_0}{1}}{\binom{A_0}{1}\binom{B_0}{1} + \binom{A_1}{1}\binom{B_0}{1} + \binom{A_0}{1}\binom{B_1}{1} + \binom{A_1}{1}\binom{B_1}{1}} = \frac{A_0.B_0}{(A_0 + A_1)(B_0 + B_1)}$$

$$P(R2) = \frac{\binom{A_1}{1}\binom{B_0}{1}}{\binom{A_0}{1}\binom{B_0}{1} + \binom{A_1}{1}\binom{B_0}{1} + \binom{A_0}{1}\binom{B_1}{1} + \binom{A_1}{1}\binom{B_1}{1}} = \frac{A_1.B_0}{(A_0 + A_1)(B_0 + B_1)}$$

$$P(R3) = \frac{\binom{A_0}{1}\binom{B_1}{1}}{\binom{A_0}{1}\binom{B_0}{1} + \binom{A_1}{1}\binom{B_0}{1} + \binom{A_0}{1}\binom{B_1}{1} + \binom{A_1}{1}\binom{B_1}{1}} = \frac{A_0.B_1}{(A_0 + A_1)(B_0 + B_1)}$$
$$P(R4) = \frac{\binom{A_1}{1}\binom{B_1}{1}}{\binom{A_0}{1}\binom{B_0}{1} + \binom{A_1}{1}\binom{B_0}{1} + \binom{A_0}{1}\binom{B_1}{1} + \binom{A_1}{1}\binom{B_1}{1}} = \frac{A_1.B_1}{(A_0 + A_1)(B_0 + B_1)}. \quad (2)$$

Due to the consumption of participating molecules after firing each reaction the probabilities of firing reactions change. However, since the reactions are symmetric, the probabilities remain proportional until the system reaches equilibrium. Only the last reaction produces C_1 and the other ones produce C_0. Therefore, the probability of generating C_1 is equal to $P(R4)$ and can be represented as follows.

$$P(C_1) = P(R4) = \frac{A_1.B_1}{(A_0 + A_1)(B_0 + B_1)} = a.b. \quad (3)$$

Since the system has only two outputs C_0 and C_1, we can write

$$P(C_1) = \frac{C_1}{(C_0 + C_1)} = c. \quad (4)$$

From (3) and (4) we can show that

$$c = a.b. \quad (5)$$

Another interpretation is to say the probability of generating a molecule of C_1 is the probability of reacting a molecule of A_1 and a molecule of B_1. Therefore, we have

$$P(C_1) = P(A_1).P(B_1) \Rightarrow \frac{C_1}{(C_0 + C_1)} = \frac{A_1}{(A_0 + A_1)}.\frac{B_1}{(B_0 + B_1)}. \quad (6)$$

We perform a Monte Carlo simulation to verify the validity and accuracy of the proposed molecular multiplier presented in Fig. 1(a) of the main text. We considered the initial quantities of molecules as $[A_0] = 30$, $[A_1] = 70$, $[B_0] = 20$, $[B_1] = 80$, and $[C_0] = [C_1] = 0$. The simulation was repeated 10^6 times. Fig. S.1.1 illustrates the simulation results. The horizontal axis in this figure represents number of molecules and vertical axis shows the number of iterations that the simulation ended with molecules C_0 and C_1.

The values of a and b are 0.7 and 0.8, respectively, based on the initial values for molecules A_0, A_1, B_0, and B_1. The simulation results show that the mean values for C_0 and C_1 are 44 and 56, respectively. Thus, the computed c is equal to 0.56. We repeat the simulation for different initial values of A_0, A_1, B_0, and B_1. Obtained mean values for C_0 and C_1 are listed in Table S.1.1.

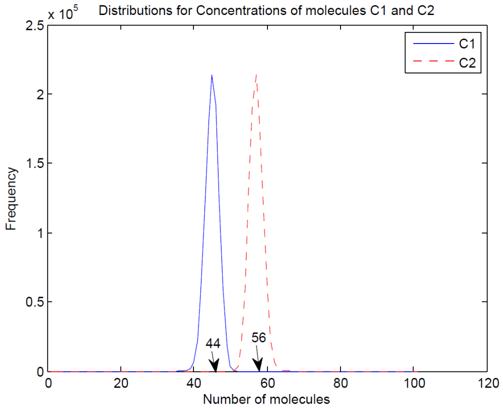

Figure S.1.1: **Monte Carlo simulation results for Mult unit.** We initialize molecules as $[A_0] = 30$, $[A_1] = 70$, $[B_0] = 20$, $[B_1] = 80$, and $[C_0] = [C_1] = 0$, and randomly fire each reaction until it can no longer be fired. We repeat the same simulation for 10^6 times and plot the number of times that the simulations terminated with each number of the molecules C_0 and C_1.

Table S.1.1: Monte Carlo simulation for different initial values of a and b.

a	b	A_0	A_1	B_0	B_1	C_0	C_1	c
0.9	0.3	10	90	70	30	73	27	0.27
0.7	0.8	30	70	20	80	44	56	0.56
0.5	0.4	50	50	60	40	80	20	0.2
0.1	0.6	90	10	40	60	94	6	0.06

S.1.2 Mass-action kinetics model

In this section, we show that the continuous molecular concentration kinetics of reactions listed in Fig. 1(a) of the main text compute the product.

For these reactions the ODEs are given by:

$$\begin{aligned}
\frac{d[A_0]}{dt} &= -[A_0][B_0] - [A_0][B_1] \\
\frac{d[A_1]}{dt} &= -[A_1][B_0] - [A_1][B_1] \\
\frac{d[B_0]}{dt} &= -[A_0][B_0] - [A_1][B_0] \\
\frac{d[B_1]}{dt} &= -[A_0][B_1] - [A_1][B_1]
\end{aligned}$$

$$\begin{aligned}\frac{d[C_0]}{dt} &= [A_0][B_0] + [A_0][B_1] + [A_1][B_0] \\ \frac{d[C_1]}{dt} &= [A_1][B_1]. \end{aligned} \tag{7}$$

It should be noted that, in our notation, $[A]$ represents the time-varying concentration of molecule A and we use it for the mass-action kinetics model.

Using the ODE equations in (7) we can prove that the CRN for Mult unit computes the multiplication in fractional encoding. We rewrite the first four equations of (7) as

$$\begin{aligned}\frac{d[A_0]}{A_0} &= -([B_0] + [B_1])dt \\ \frac{d[A_1]}{A_1} &= -([B_0] + [B_1])dt \\ \frac{d[B_0]}{B_0} &= -([A_0] + [A_1])dt \\ \frac{d[B_1]}{B_1} &= -([A_0] + [A_1])dt. \end{aligned} \tag{8}$$

Comparing the first two equations of (8) we have

$$\int_0^t \frac{d[A_0]}{[A_0]} = \int_0^t \frac{d[A_1]}{[A_1]} = \int_0^t ([B_0] + [B_1])dt \tag{9}$$

Suppose a_0 and a_1,respectively, represent the initial concentrations for the molecules A_0 and A_1. From (9) we have

$$\begin{aligned}\ln[A_0] - \ln a_0 = \ln[A_1] - \ln a_1 \\ \Rightarrow \tfrac{[A_0]}{a_0} = \tfrac{[A_1]}{a_1}. \end{aligned} \tag{10}$$

Similarly, from the last two reactions in (8) we obtain

$$\frac{[B_0]}{b_0} = \frac{[B_1]}{b_1} \tag{11}$$

where b_0 and b_1 are the initial concentrations for the molecules B_0 and B_1, respectively. The initial values for molecules C_0 and C_1 are zero and we can write

$$\frac{[C_1]}{[C_0] + [C_1]} = \frac{\frac{d[C_1]}{dt}}{\frac{d[C_0]}{dt} + \frac{d[C_1]}{dt}}. \tag{12}$$

From the last two equations of (7) we write

$$\frac{[C_1]}{[C_0]+[C_1]} = \frac{[A_1][B_1]}{[A_0][B_0]+[A_0][B_1]+[A_1][B_0]+[A_1][B_1]}. \tag{13}$$

By inserting (10) and (11) we obtain

$$\begin{aligned} c = \frac{[C_1]}{[C_0]+[C_1]} &= \frac{[A_1][B_1]}{\frac{a_0 b_0}{a_1 b_1}[A_1][B_1] + \frac{a_0}{a_1}[A_1][B_1] + \frac{b_0}{b_1}[A_1][B_1] + [A_1][B_1]} \\ &= \frac{[A_1][B_1]}{[A_1][B_1](\frac{a_0 b_0}{a_1 b_1} + \frac{a_0}{a_1} + \frac{b_0}{b_1} + 1)} \\ &= \frac{1}{\frac{a_0 b_0}{a_1 b_1} + \frac{a_0}{a_1} + \frac{b_0}{b_1} + 1} \\ &= \frac{a_1 b_1}{(a_0+a_1)(b_0+b_1)} = a \times b. \end{aligned} \tag{14}$$

Note that in Equation (14) we assume that molecular types A_0, A_1, B_0, and B_1 are initialized such that $a = \frac{a_1}{(a_0+a_1)}$ and $b = \frac{b_1}{(b_0+b_1)}$.

S.2 Variations of Mult units

The CRN for the multiplication module can be modified such that the concentrations of molecules related to one of the inputs remains unchanged. The set of molecular reactions that preserves molecules of A_0 and A_1 is shown in (15). Similar to the CRN presented in Fig. 1(a) of the main text, it is easy to show that the CRN listed in (15) computes the product $c = a \times b$ in fractional coding.

$$\begin{aligned} A_0 + B_0 &\rightarrow C_0 + A_0 \\ A_0 + B_1 &\rightarrow C_0 + A_0 \\ A_1 + B_0 &\rightarrow C_0 + A_1 \\ A_1 + B_1 &\rightarrow C_1 + A_1. \end{aligned} \tag{15}$$

Furthermore, the CRN can be designed such that the concentration of both input molecules remain unchanged while they produce the output molecules. In order to avoid the infinite output concentrations, we add two more reactions for annihilation of the output molecules. The set of reactions in (16) shows the CRN for this module.

$$\begin{aligned} A_0 + B_0 &\rightarrow C_0 + A_0 + B_0 \\ A_0 + B_1 &\rightarrow C_0 + A_0 + B_1 \end{aligned}$$

$$
\begin{aligned}
A_1 + B_0 &\rightarrow C_0 + A_1 + B_0 \\
A_1 + B_1 &\rightarrow C_1 + A_1 + B_1 \\
C_0 &\rightarrow \varnothing \\
C_1 &\rightarrow \varnothing.
\end{aligned} \tag{16}
$$

For these reactions the ODEs are given by:

$$
\begin{aligned}
\frac{d[C_0]}{dt} &= [A_0][B_0] + [A_0][B_1] + [A_1][B_0] - [C_0] \\
\frac{d[C_1]}{dt} &= [A_1][B_1] - [C_1].
\end{aligned} \tag{17}
$$

Then at the equilibrium we have

$$
\begin{aligned}
[C_0] &= [A_0][B_0] + [A_0][B_1] + [A_1][B_0] \\
[C_1] &= [A_1][B_1].
\end{aligned} \tag{18}
$$

Therefore the output value, c, is given by:

$$
\begin{aligned}
c = \frac{[C_1]}{[C_0] + [C_1]} &= \frac{[A_1][B_1]}{[A_0][B_0] + [A_0][B_1] + [A_1][B_0] + [A_1][B_1]} \\
&= \frac{[A_1]}{[A_0] + [A_1]} \frac{[B_1]}{[B_0] + [B_1]} = ab.
\end{aligned} \tag{19}
$$

Clearly, the alternative CRNs for NMult unit can be designed in a similar way.

S.3 Proof for Bipolar Mult Unit

Similar to the Mult for unipolar fractional coding and using both stochastic modeling and mass-action kinetics, in this section we prove that the Mult unit computes the product of its inputs in bipolar fractional representation.

S.3.1 Stochastic model:

In bipolar fractional coding we have

$$
c = \frac{C_1 - C_0}{C_0 + C_1} = \frac{C_1}{C_0 + C_1} - \frac{C_0}{C_0 + C_1} = P(C_1) - P(C_0). \tag{20}
$$

It means that the output c is equal to the probability of producing C_1 minus the probability of producing C_0. Since the first and last reactions of the bipolar Mult produce C_1 and the other

two reactions produce C_0, according to the stochastic modeling of chemical reaction networks, these probabilities are computed as:

$$P(C_0) = \frac{A_0B_1 + A_1B_0}{(A_0 + A_1)(B_0 + B_1)} \tag{21}$$

$$P(C_1) = \frac{A_0B_0 + A_1B_1}{(A_0 + A_1)(B_0 + B_1)}. \tag{22}$$

Therefore, we have

$$\begin{aligned} c &= \frac{A_0B_0 + A_1B_1}{(A_0 + A_1)(B_0 + B_1)} - \frac{A_0B_1 + A_1B_0}{(A_0 + A_1)(B_0 + B_1)} \\ &= \frac{(A_1 - A_0)(B_1 - B_0)}{(A_0 + A_1)(B_0 + B_1)} = a \times b. \end{aligned} \tag{23}$$

S.3.2 Mass-action kinetics model:

The proof based on the Mass-action kinetics model for bipolar fractional coding is similar to the one for the unipolar fractional coding. The first four ODEs in (7) and the two ODEs in (24) constitute the ODEs for the molecular reactions of the bipolar Mult unit.

$$\begin{aligned} \frac{d[C_0]}{dt} &= [A_0][B_1] + [A_1][B_0] \\ \frac{d[C_1]}{dt} &= [A_0][B_0] + [A_1][B_1]. \end{aligned} \tag{24}$$

Assuming the initial concentrations of C_0 and C_1 are zero, from the equations in (24) we have

$$\begin{aligned} c &= \frac{[C_1] - [C_0]}{[C_0] + [C_1]} = \frac{\frac{d[C_1]}{dt} - \frac{d[C_0]}{dt}}{\frac{d[C_0]}{dt} + \frac{d[C_1]}{dt}} \\ &= \frac{[A_0][B_0] + [A_1][B_1] - [A_0][B_1] - [A_1][B_0]}{([A_0] + [A_1])([B_0] + [B_1])}. \end{aligned} \tag{25}$$

Using (10) and (11), earlier obtained from the first four ODEs in (7), we have

$$c = \frac{[A_1][B_1](\frac{a_0}{a_1}\frac{b_0}{b_1} + 1 - \frac{b_0}{b_1} - \frac{a_0}{a_1})}{[A_1][B_1](\frac{a_0}{a_1}\frac{b_0}{b_1} + 1 + \frac{b_0}{b_1} + \frac{a_0}{a_1})} = \frac{(a_1 - a_0)(b_1 - b_0)}{(a_1 + a_0)(b_1 + b_0)} = a \times b. \tag{26}$$

Obviously, we assumed that molecular types A_0, A_1, B_0, and B_1 are initialized such that $a = \frac{a_1 - a_0}{(a_0 + a_1)}$ and $b = \frac{b_1 - b_0}{(b_0 + b_1)}$.

The proof for bipolar NMult unit is similar to the proof for the unipolar Mult unit.

S.4 Proof for the MUX Unit

The MUX unit is implemented using the four reactions shown in Fig. 1(c) of the main text. These reactions compute scaled addition both for unipolar and bipolar fractional representations. We show this based on the stochastic and mass-action kinetics models.

S.4.1 Stochastic model

As described earlier for the stochastic model we compute the probabilities of firing reactions. The probability of producing C_1 is given by Equation (27).

$$c = \frac{C_1}{C_0 + C_1} = P(C_1) = \frac{A_1 S_0 + B_1 S_1}{(A_0 + A_1)S_0 + (B_0 + B_1)S_1}. \tag{27}$$

If $(A_0 + A_1) = (B_0 + B_1)$, Equation (27) can be rewritten as

$$c = \frac{A_1 S_0}{(A_0 + A_1)(S_0 + S_1)} + \frac{B_1 S_1}{(B_0 + B_1)(S_0 + S_1)} = (1 - s)a + sb. \tag{28}$$

Note that (A_0+A_1) must equal (B_0+B_1) during the initial selection of reactant concentrations. For bipolar fractional coding we compute $P(C_1) - P(C_0)$ as follows:

$$c = \frac{C_1 - C_0}{C_0 + C_1} = P(C_1) - P(C_0) = \frac{(A_1 S_0 + B_1 S_1) - (A_0 S_0 + B_0 S_1)}{(A_0 + A_1)S_0 + (B_0 + B_1)S_1}. \tag{29}$$

Since $(A_0 + A_1) = (B_0 + B_1)$, we can rewrite Equation (29) as given in Equation (30).

$$c = \frac{(A_1 - A_0)S_0}{(A_0 + A_1)(S_0 + S_1)} + \frac{(B_1 - B_0)S_1}{(B_0 + B_1)(S_0 + S_1)} = (1 - s)a + sb. \tag{30}$$

S.4.2 Mass-action kinetics model

For molecules of C_0 and C_1, the ODEs of chemical reactions shown in Fig. 1(c) are described by Equation (31).

$$\begin{aligned} \frac{d[C_0]}{dt} &= [A_0][S_0] + [B_0][S_1] \\ \frac{d[C_1]}{dt} &= [A_1][S_0] + [B_1][S_1]. \end{aligned} \tag{31}$$

By assuming that the initial concentration for C_0 and C_1 is zero, for unipolar fractional coding the output c can be computed by Equation (32).

$$c = \frac{[C_1]}{[C_0] + [C_1]} = \frac{\frac{d[C_1]}{dt}}{\frac{d[C_0]}{dt} + \frac{d[C_1]}{dt}}$$

$$= \frac{[A_1][S_0] + [B_1][S_1]}{([A_0] + [A_1])[S_0] + ([B_0] + [B_1])[S_1]}. \quad (32)$$

If $([A_0] + [A_1]) = ([B_0] + [B_1])$ we can rewrite Equation (32) as given by Equation (33).

$$c = \frac{[A_1][S_0]}{([A_0] + [A_1])([S_0] + [S_1])} + \frac{[B_1][S_1]}{([B_0] + [B_1])([S_0] + [S_1])} = (1 - s)a + sb. \quad (33)$$

Similarly for bipolar fractional coding we can compute c by Equation (34).

$$\begin{aligned} c &= \frac{[C_1] - [C_0]}{[C_0] + [C_1]} = \frac{\frac{d[C_1]}{dt} - \frac{d[C_0]}{dt}}{\frac{d[C_0]}{dt} + \frac{d[C_1]}{dt}} \\ &= \frac{[A_1][S_0] + [B_1][S_1] - ([A_1][S_1] + [B_0][S_1]}{([A_0] + [A_1])[S_0] + ([B_0] + [B_1])[S_1]}. \end{aligned} \quad (34)$$

If $([A_0] + [A_1]) = ([B_0] + [B_1])$, we can rewrite Equation (34) as given by Equation (35).

$$c = \frac{([A_1] - [A_0])[S_0]}{([A_0] + [A_1])([S_0] + [S_1])} + \frac{([B_1] - [B_0])[S_1]}{([B_0] + [B_1])([S_0] + [S_1])} = (1 - s)a + sb. \quad (35)$$

S.5 Molecular Inner Product

The set of eight reactions listed in (36) computes the scaled version of inner product for two vectors $\begin{bmatrix} a \\ c \end{bmatrix}$ and $\begin{bmatrix} b \\ d \end{bmatrix}$ in unipolar fractional coding.

$$\begin{aligned} A_0 + B_0 &\rightarrow E_0 & C_0 + D_0 &\rightarrow E_0 \\ A_0 + B_1 &\rightarrow E_0 & C_0 + D_1 &\rightarrow E_0 \\ A_1 + B_0 &\rightarrow E_0 & C_1 + D_0 &\rightarrow E_0 \\ A_1 + B_1 &\rightarrow E_1 & C_1 + D_1 &\rightarrow E_1. \end{aligned} \quad (36)$$

According to the stochastic model we have

$$e = \frac{E_1}{E_0 + E_1} = P(E_1) = \frac{A_1B_1 + C_1D_1}{(A_0 + A_1)(B_0 + B_1) + (C_0 + C_1)(D_0 + D_1)}. \quad (37)$$

If $([A_0] + [A_1])([B_0] + [B_1]) = (C_0 + C_1)(D_0 + D_1)$ we can rewrite Equation (37) as given by Equation (38).

$$e = \frac{A_1B_1 + C_1D_1}{2(A_0 + A_1)(B_0 + B_1)} = \frac{1}{2}(ab + cd). \quad (38)$$

In general, we can compute the inner product of two $n \times 1$ vectors using $4n$ chemical reactions where each of the four reactions is related to a corresponding pair of elements of the input vectors. In fact, if $x = \begin{bmatrix} x_1 \\ x_2 \\ : \\ x_N \end{bmatrix}$ and $w = \begin{bmatrix} w_1 \\ w_2 \\ : \\ w_N \end{bmatrix}$ are input vectors, the proposed molecular reactions compute the scaled version of their inner product as shown in Equation (39).

$$y = \frac{1}{N}(x_1 w_1 + x_2 w_2 + ... + x_N w_N) \tag{39}$$

Evidently, similar to the unipolar case for bipolar coding the set of eight reactions in (40) computes the scaled inner product of two vectors $\begin{bmatrix} a \\ c \end{bmatrix}$ and $\begin{bmatrix} b \\ d \end{bmatrix}$. In other words reactions listed in (40) compute $\frac{1}{2}(ab + cd)$ in bipolar fractional coding.

$$\begin{aligned} A_0 + B_0 &\rightarrow E_1 \qquad & C_0 + D_0 &\rightarrow E_1 \\ A_0 + B_1 &\rightarrow E_0 & C_0 + D_1 &\rightarrow E_0 \\ A_1 + B_0 &\rightarrow E_0 & C_1 + D_0 &\rightarrow E_0 \\ A_1 + B_1 &\rightarrow E_1 & C_1 + D_1 &\rightarrow E_1 \end{aligned} \tag{40}$$

S.6 Maclaurin Series Expansion of Target Functions

Maclaurin series expansion of target functions are listed below.

$$e^{-x} = \sum_{n=0}^{\infty} \frac{(-x)^n}{n!} = 1 - x + \frac{x^2}{2!} - \frac{x^3}{3!} + \frac{x^4}{4!} - ... \tag{41}$$

$$\sin(x) = \sum_{n=0}^{\infty} \frac{(-1)^n}{(2n+1)!} x^{(2n+1)} = x - \frac{x^3}{3!} + \frac{x^5}{5!} - ... \tag{42}$$

$$\cos(x) = \sum_{n=0}^{\infty} \frac{(-1)^n}{(2n)!} x^{(2n)} = 1 - \frac{x^2}{2!} + \frac{x^4}{4!} - .... \tag{43}$$

$$\log(1+x) = \sum_{n=0}^{\infty} (-1)^{n+1} \frac{x^n}{n!} = x - \frac{x^2}{2} + \frac{x^3}{3} - \frac{x^4}{4} + ... \tag{44}$$

$$\tanh(x) = \sum_{n=1}^{\infty} \frac{B_{2n} 4^n (4^n - 1)}{(2n)!} x^{2n-1} = x - \frac{1}{3}x^3 + \frac{2}{15}x^5 - \frac{17}{315}x^7 + ... \tag{45}$$

$$\text{sigmoid}(x) = \frac{1}{1+e^{-x}} = \sum_{n=0}^{\infty} \frac{(-1)^n E_n(0)}{(2n)!} x^n = 1 - \frac{1}{2} + \frac{x}{4} - \frac{x^3}{48} + \frac{x^5}{480} + ... \tag{46}$$

$$\frac{sin(\pi x)}{\pi} = \sum_{n=0}^{\infty} \frac{(-1)^n}{(2n+1)!} \pi^{2n} x^{(2n+1)} = x - \frac{\pi^2 x^3}{3!} + \frac{\pi^4 x^5}{5!} - \frac{\pi^6 x^7}{7!} + \frac{\pi^8 x^9}{9!} - ... \tag{47}$$

$$cos(\pi x) = \sum_{n=0}^{\infty} \frac{(-1)^n}{(2n)!} (\pi x)^{(2n)} = 1 - \frac{(\pi x)^2}{2!} + \frac{(\pi x)^4}{4!} - \frac{(\pi x)^6}{6!} + \frac{(\pi x)^8}{8!} - ... \tag{48}$$

where B_n is a Bernoulli number, $E_n(x)$ is an Euler polynomial, and for logarithm function $|x| \leq 1$.

S.7 Coefficients and Inputs for Perceptron

	A	B	C
w_1	0.5	0.5	0.5
w_2	0.5	0.5	0.5
w_3	0.5	0.5	0.5
w_4	0.5	0.5	0.5
w_5	0.5	0.5	0.5
w_6	0.5	0.5	0.5
w_7	0.5	0.5	-0.5
w_8	0.5	0.5	-0.5
w_9	-0.5	0.5	-0.5
w_{10}	-0.5	0.5	-0.5
w_{11}	-0.5	-0.5	-0.5
w_{12}	-0.5	-0.5	-0.5
w_{13}	-0.5	-0.5	-0.5
w_{14}	-0.5	-0.5	-0.5
w_{15}	-0.5	-0.5	-0.5
w_{16}	-0.5	-0.5	-0.5
w_{17}	0.25	0.25	0.25
w_{18}	0.25	0.25	0.25
w_{19}	0.25	0.25	0.25
w_{20}	0.25	0.25	0.25
w_{21}	0.25	0.25	0.25
w_{22}	0.25	0.25	0.25
w_{23}	0.25	0.25	-0.25

w_{24}	0.25	0.25	-0.25
w_{25}	-0.25	0.25	-0.25
w_{26}	-0.25	0.25	-0.25
w_{27}	-0.25	-0.25	-0.25
w_{28}	-0.25	-0.25	-0.25
w_{29}	-0.25	-0.25	-0.25
w_{30}	-0.25	-0.25	-0.25
w_{31}	-0.25	-0.25	-0.25
w_{32}	-0.25	-0.25	-0.25

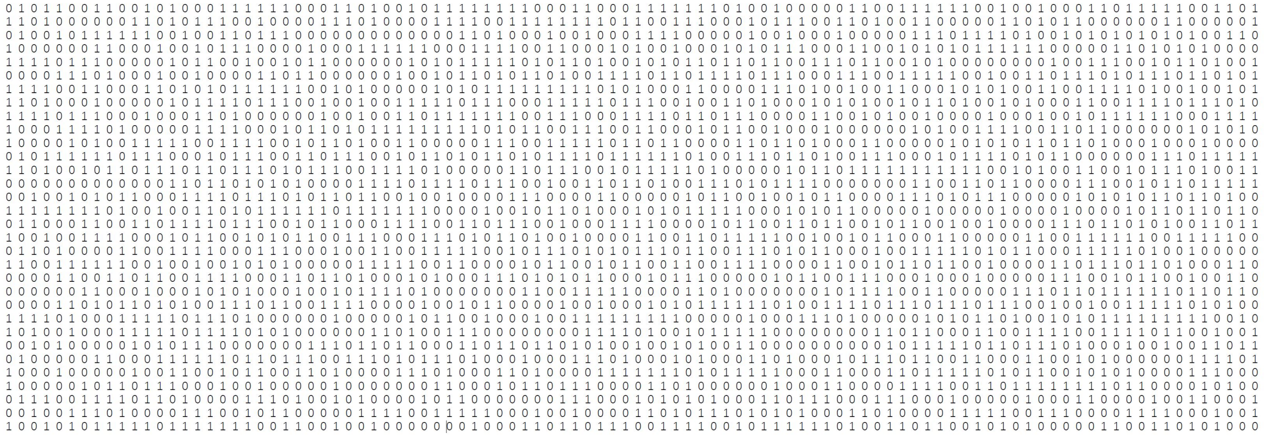

Figure S.7.1: **Inputs of three perceptrons, denoted A, B and C.** Inputs to the perceptron: each column of the 32×100 matrix illustrates an input vector containing 32 binary inputs.

S.8 DNA Implementation Details

The proposed CRNs for computing functions are composed of bimolecular reactions with one product. We use the template presented in Fig. S.8.1 for implementation of our molecular reactions by DSD reactions.

Fig. S.8.1 shows a sequence of six DNA reactions, R1-R6, that implement molecular reaction $A + B \rightarrow C$. All DNA reactions are based on the toehold mediated mechanism first presented in [2]. The primary molecules, A, B, and C, are represented by single strand DNA molecules – red strands in Fig. S.8.1 – composed of a toehold and a main domain part. The initial system provides required gate and auxiliary molecules, i.e., DNA molecules G1, G2, <tr r>, <c tr>, and <i tc> – black strands in Fig. S.8.1 – are initially available in the system. Furthermore, the concentration of gate and auxiliary strands are initialized to be large enough to efficiently supply the sequence of DNA reactions to continue as long as the primary molecules last.

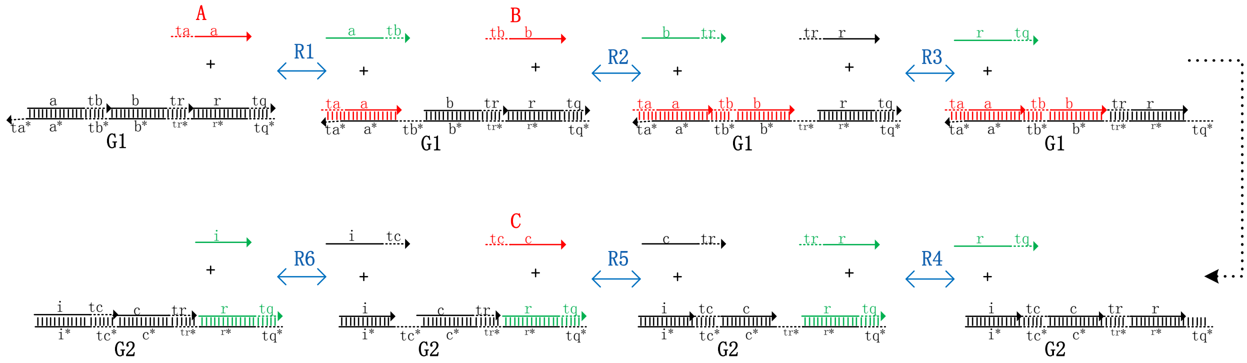

Figure S.8.1: **DNA implementation of $A+B \rightarrow C$.** According to the methodology developed in [1], a sequence of six DNA strand displacement reactions, $R1 - R6$, implement bimolecular reaction $A + B \rightarrow C$.

Each reaction in the sequence of DNA reactions produces the mediating toehold for the next reaction. The sequence starts when the toehold domain of input molecule A, i.e., ta, binds with its WatsonCrick complementary domain in gate G1, i.e., ta*. This leads to the binding of whole molecules of A to gate G1. Similarly, through reaction R2, the DNA molecule B binds to gate G1 and in Reaction R5 the output DNA molecule C is released from gate G2. For the details about the mechanism, the reader is referred to [1]. The authors in [1] have experimentally validated that the sequence of DNA strand displacement reactions in Fig S.8.1 does implement the expected kinetics for the desired bimolecular reaction. They also showed that the rate constant can be tuned by adjusting the initial concentrations of gates and auxiliary molecules. The linear, double-stranded DNA molecules used in the mechanism can be derived from biologically synthesized (plasmid) DNA. Compatibility with natural DNA leads to the reduction of errors associated with chemically synthesized DNA.

For each of the six target functions in this paper we perform the DNA simulation based on the software provided in [3]. Each function is computed for 11 different inputs and the results are demonstrated in Fig. S.8.2. The DNA computed outputs are shown by red stars and the exact values of functions are shown as blue lines. The DNA computed values follow the exact values with an acceptable accuracy for all functions except $\frac{sin(\pi x)}{\pi}$, $\frac{cos(\pi x)}{5.9348}$ and $sigmoid(x)$ for large values of x. The molecular outputs for these three functions cannot reach steady-state values within 50 hours of simulation. For example, at $x = 0.9$, the DNA simulation (exact) values of these three functions are given by, 0.1329 (0.0984), -0.0852 (-0.1603), and 0.6906 (0.7109), respectively, after 50 hours of simulation time. However, these DNA reactions can reach more accurate values after longer simulation time. Figure S.8.3 shows the kinetics of the DNA simulations for these three functions for $x = 0.9$. The DNA simulation (exact) values of

these functions at $x = 0.9$ are given by 0.1023 (0.0984), -0.1416 (-0.1603), and0.7061 (0.7109) after 200, 200 and 500 hours of simulation time, respectively. These DNA outputs are closer to exact values.

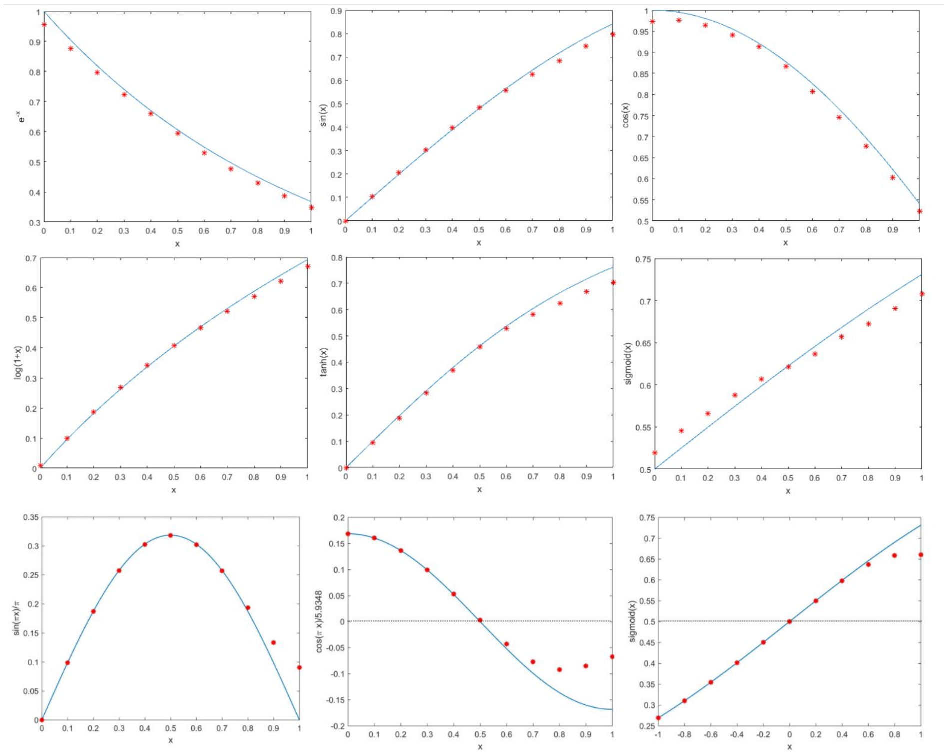

Figure S.8.2: **Exact and computed values of the functions.** Computed values of functions using our proposed molecular systems along their exact graphs for e^{-x}, $\sin(x)$, $\cos(x)$, $\log(1+x)$, $\tanh(x)$, and sigmoid(x), $\frac{sin(\pi x)}{\pi}$, and $\frac{cos(\pi x)}{5.9348}$ with unipolar input, and sigmoid(x) with bipolar input. For $\frac{cos(\pi x)}{5.9348}$, the output is in bipolar encoding. Blue lines: exact values, red stars: computed values.

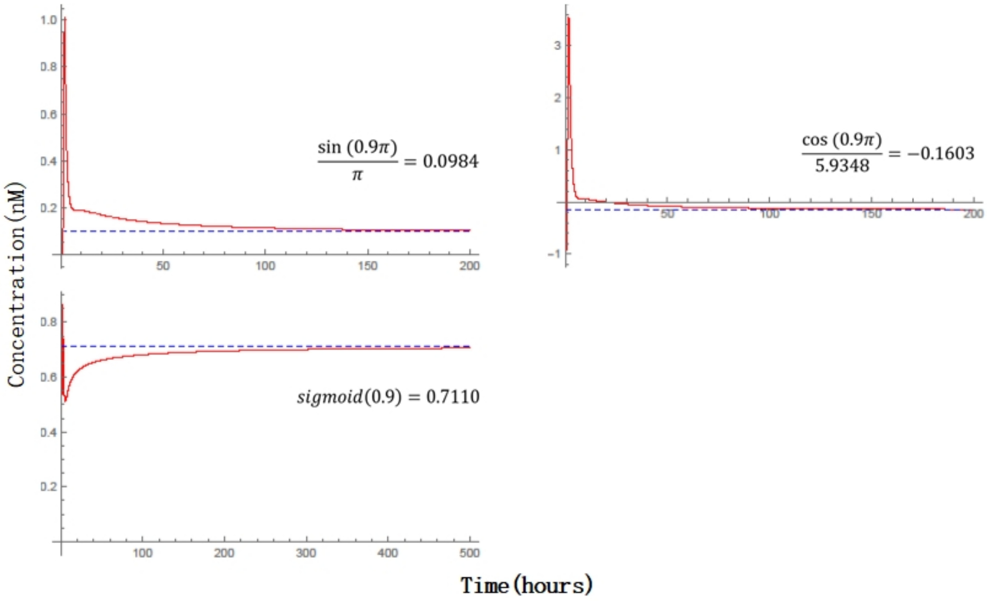

Figure S.8.3: **DNA simulation results.** The DNA reaction kinetics for the computation of $\frac{sin(\pi x)}{\pi}$, and $\frac{cos(\pi x)}{5.9348}$ and sigmoid(x) for x = 0.9.

References

[1] Chen, Y.J., Dalchau, N., Srinivas, N., Phillips, A., Cardelli, L., Soloveichik, D. & Seelig, G. Programmable Chemical Controllers Made from DNA. Nature Nanotechnology **8,** 755-762 (2013).

[2] Yourke, B., Turberfield, A. J., Mills, A. P., Simmel, F. C.,& Neumann, J. L. A DNA-fuelled Molecular Machine Made of DNA. Nature **406,** 605-608 (2000).

[3] Soloveichik, D., Seelig, G. & Winfree, E. DNA as a Universal Substrate for Chemical Kinetics. Proceedings of the National Academy of Sciences (PNAS), 5393–5398 (2010).
